# Process Intensification for the Synthesis of 6-Allyl-6-azabicyclo[3.1.0]hex-3-en-2-ol from 1-Allylpyridinium Salt Using a Continuous UV-Light Photoflow Approach

**DOI:** 10.3390/mps2030067

**Published:** 2019-08-05

**Authors:** Milene A. G. Fortunato, Chi-Phong Ly, Filipa Siopa, Carlos A. M. Afonso

**Affiliations:** 1 Research Institute for Medicines (iMed.ULisboa), Faculty of Pharmacy, Universidade de Lisboa, Av. Prof. Gama Pinto, 1649-003 Lisboa, Portugal; 2 Sorbonne Université, Faculté des Sciences et Ingénierie, CNRS, Institut Parisien de Chimie Moléculaire, IPCM, 4 Place Jussieu, 75005 Paris, France

**Keywords:** flow reaction, photochemistry, bicyclic aziridine

## Abstract

A new home-made UV photochemical reactor (95 cm of irradiation zone) consisting of a 12 parallel quartz tubes flow reactor, PQT6 (95 cm under irradiation and an internal diameter of 0.6 cm) was assembled to perform photochemical transformations in continuous-flow. PQT6 was evaluated for the photoreaction of 1-allylpyridinium bromide (**1a**) to 6-allyl-6-azabicyclo[3.1.0]hex-3-en-2-ol (**2a**), in a continuous process. This technology provides reduced reaction times, continuous production of **2a**, and a productivity of 129 mg h^−1^, corresponding to 1.94 g of isolated **2a** after 15 h of irradiation.

## 1. Introduction

**Scheme 1:**
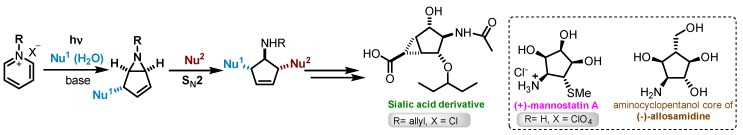
General synthetic pathway for aminocyclopentitols *via* photoreaction of pyridinium salts [1,2,3,4].

Bicyclic aziridines (α-hydroxycyclopenteno-aziridines) prepared by photochemical transformation of pyridinium salts are key intermediates used for the total synthesis of several aminocyclopentitols (Scheme 1) [1,2,3,4]. In 2016, Bennet and co-workers, described the synthesis of sialic acid derivatives using this synthetic methodology. Under batch conditions, 1-allylpyridinium chloride was irradiated during 16 h to afford 630 mg (60% yield) of 6-allyl-6-azabicyclo[3.1.0]hex-3-en-2-ol (**2a**), with a productivity of 39.4 mg h^−1^ [5].

**Figure 1:**
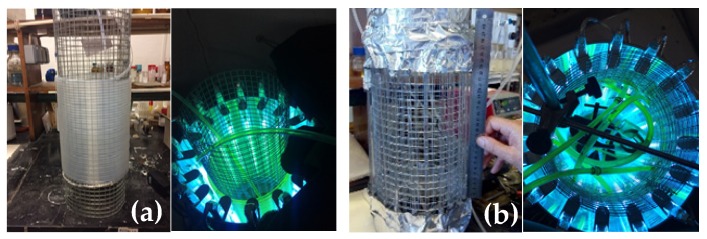
Previously reported home-made continuous-flow reactors. (**a**) FEP4 reactor [(2296 cm (*l*) × 0.4 cm (*d*)] around metal grid (left), and top-view under irradiation (right). (**b**) Parallel quartz tube reactor (PQT4), [24 tubes in vertical orientation and parallel to each other: 22.5 cm (*l*) × 0.4 cm (*d*)] (left) and top-view under irradiation (right) [7].

Recently, we developed different types of home-made continuous-flow reactors to overcome the reduced productivity problem of this photoreaction (Scheme 1). A fluorinated ethylene propylene (FEP4) tube reactor (internal diameter of 0.4 cm, Figure 1a) similar to that described by Booker-Milburn [6], and two parallel quartz tube (PQT) reactors (internal diameters 0.4 cm denoted as PQT4, Figure 1b; and 0.2 cm) were used [7].

**Scheme 2:**
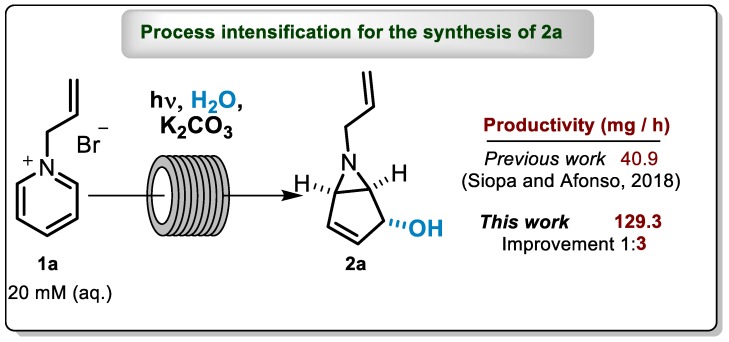
Synthetic methodology for the synthesis of **2a**.

The reactors were assessed for the photoflow transformation of 1-allyl and 1-n-butylpyridinium salts to the correspondent bicyclic aziridines, under recirculating (FEP4, PTQ4 and PTQ2) and continuous-flow conditions (FEP4) [7]. Under recirculating conditions, PQT4 gave the best productivity results for both bicyclic aziridines. Furthermore, regarding the isolated 6-allyl-6-azabicyclo[3.1.0]hex-3-en-2-ol, (**2a**) (Scheme 2) and 6-butyl-6-azabicyclo[3.1.0]hex-3-en-2-ol (**2b**), this reactor was capable of producing 40.9 mg h^−1^ and 87.0 mg h^−1^, respectively [7], as highlighted by Snieckus, V. and Allais, C. in Synfact in 2018 [8]. Despite these outstanding productivity results, the PQT4 reactor presents some drawbacks as a consequence of its design, such as a large dead-volume, resulting from the connections between the different quartz tubes (Figure 1b). Such limitation has so far prevented the use of PQT4 reactor in a continuous process. 

**Figure 2:**
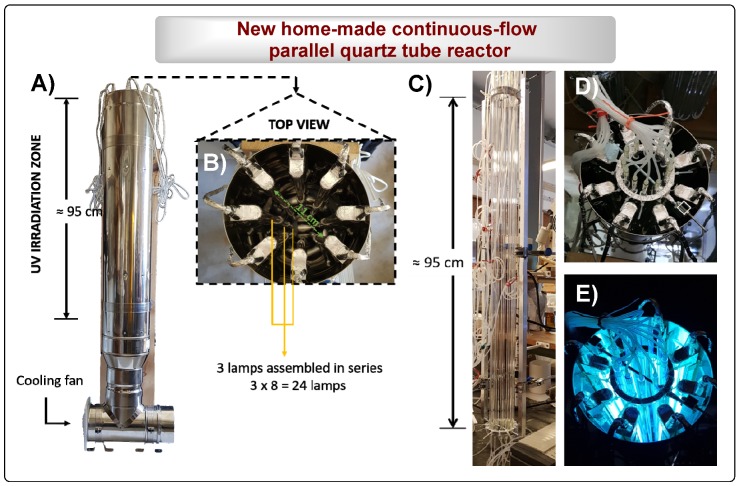
New home-made UV reactor and continuous-flow parallel quartz tube reactor. (**A**) Overview of the new home-made UV reactor; (**B**) UV reactor top-view; (**C**) 12 assembled parallel quartz tubes reactor (PQT6); (**D**) Reactors top-view; (**E**) Top-view of the reactors under photochemical irradiation.

To overcome these limitations, herein is presented the development of a home-made UV reactor and a PQT reactor capable of operating under continuous-flow. The reactors were designed for higher scale production of **2a** *via* photochemical irradiation of **1a** (Scheme 2 and Figure 2).

## 2. Experimental Design

**Figure 3:**
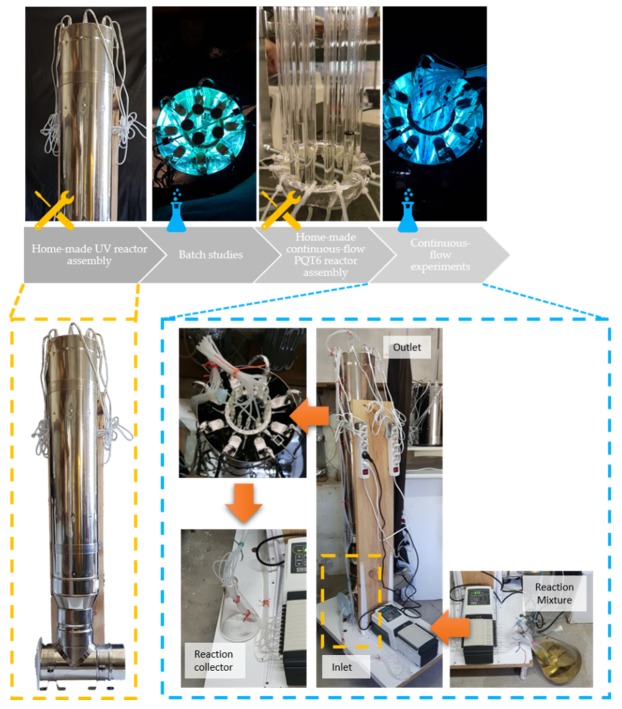
Workflow overview.

The workflow for the process intensification of 6-allyl-6-azabicyclo[3.1.0]hex-3-en-2-ol (**2a**) *via* photochemical irradiation of 1-allylpyridinium bromide (**1a**) is divided into four stages: (i) home-made UV reactor assembly, (ii) batch studies, (iii) home-made continuous-flow PQT reactor assembly and (iv) continuous-flow experiments, as shown in Figure 3.

### 2.1. Materials

- Pyridine (Carlo Erba, CAS no. 110-86-1, Product Sku 469622)
- Allyl bromide (Sigma-Aldrich (97%), CAS no. 106-95-6)
- Potassium carbonate (Sigma-Aldrich (99%), CAS no. 584-08-7)
- Dichloromethane (Valentim Ribeiro 99–100%, Ref. VR0370)

### 2.2. Equipment

- Home-made UV reactor containing 24 PURITEC HNS Germicidal Lamps Ref. HNS 8W G5 G5 (8W at 254 nm) with 144 cm length and 95 cm of irradiation zone (3 lamps assembled in line).
- Quartz tubes (QT): [total length (*l*), length under irradiation zone (*l*) and internal diameter (*d*)];

QT2: [±100 cm (*l*), 95 cm under irradiation (*l*) × 0.2 cm (*d*)];

QT4: [±100 cm (*l*), 95 cm under irradiation (*l*) × 0.4 cm (*d*)];

QT6: [±100 cm (*l*), 95 cm under irradiation (*l*) × 0.6 cm (*d*)].

- Quartz tubes’ support for the home-made UV reactor.
- Home-made continuous-flow parallel quartz tube reactor (PQT6): [12 tubes: 95 cm under irradiation (*l*) × 0.6 cm (*d*)].
- Multichannel cassette pumps Watson Marlow 205S.
- 24 silicon tubes 1 × 3 mm, of ±2 m each, Ref. 350013.

## 3. Procedure

### 3.1. General Methods

^1^H NMR spectra were obtained on a Bruker Fourier 300 spectrometer. NMR experiments were performed in D_2_O at room temperature. Chemical shifts are given in parts per million (ppm), the symbols m, s, d, t, and q represent multiplet, singlet, doublet, triplet, and quartet, respectively and the coupling constants (*J*) are given in Hertz (Hz). 1-allylpyridinium bromide (**1a**) was prepared as previously described by us [7].

### 3.2. General Procedure for Batch Photochemical Transformation of **1a** to **2a**

With the quartz tubes (QT2, QT4, QT6) in the support, an aqueous solution of **1a** with potassium carbonate (1.2 mol equiv.) in a specific concentration (20, 40, 60, or 80 mM) was added. The support was placed inside the photochemical home-made UV reactor and was irradiated up to 10 h at room temperature (the measured temperature inside the top of the reactor was below 34 ºC). At selected times, samples were collected from the reaction mixture, concentrated under vacuum and analyzed by ^1^H NMR (D_2_O). All data are available in the Supporting Information.

### 3.3. Optimization of the Reaction Conditions for Continuous-Flow Photochemical Transformation of **1a** to **2a**

#### 3.3.1. Using PQT6 with 12 QTs under Continuous-Flow Conditions

PQT6 reactor was placed inside the UV photochemical reactor. A 600 mL of an aqueous solution of **1a** (20 mM) and potassium carbonate (1.2 molar equiv.) was prepared. The solution was pumped (multichannel cassette pumps Watson Marlow 205S, using 12 silicon tubes 1 × 3 mm) onto PQT6, at 8.75 rpm which correspond to a 0.35 mL/min flow rate. After 1.3 h of irradiation, a sample was collected, concentrated and analyzed by ^1^H NMR (D_2_O).

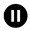
**PAUSE STEP** In order to save resources from the laboratory, the experiences described in Section 3.3.2 used 11 QTs under recirculating continuous-flow conditions, with the solution already irradiated in Section 3.3.1. These 11 tubes were not used for analysis of the reaction, they were only used to mimic the continuous operation conditions using 12 tubes.

In addition, 1 QT was removed from PQT6 reactor, cleaned and placed back in the support. This clean QT was used under continuous-flow conditions and allowed determination of the reaction conversion by ^1^H NMR.

#### 3.3.2. Using PQT6 with 1 QT under Continuous-Flow and 11 QTs under Recirculating Continuous-Flow

With PQT6 reactor already in the UV reactor, a 100 mL of fresh solution of **1a** (20 mM) and potassium carbonate (1.2 molar equiv.) was pumped towards 1 QT, using a multichannel cassette peristaltic pump (Watson Marlow 205S) at 5 rpm (0.21 mL/min flow rate, residence time 2.3 h) and in the second experience at 3.5 rpm (0.14 mL/min flow rate, residence time 3.3 h). After the respective residence time, samples were analyzed by ^1^H NMR (D_2_O).

As stated above, the remaining 11 QTs were also pumped at the same flow rate, 0.21 or 0.14 mL/min, corresponding to 5 or 3.5 rpm respectively, using the solution previous irradiated in Section 3.3.1. As these tubes are under recirculating continuous-flow conditions, they were not used for analysis.

### 3.4. General Procedure for Continuous-Flow Photochemical Transformation of **1a** to **2a**

The PQT6 reactor consisting of 12 QT6 tubes was placed inside the home-made UV reactor. To fill the PQT6, a 350 mL of an aqueous solution of **1a** (20 mM) and potassium carbonate (1.2 molar equiv.) was pumped using the multichannel cassette pumps Watson Marlow 205S (12 silicon tubes 1 × 3 mm). Then, the pump was set to 3 rpm (0.12 mL/min flow rate) and the irradiation was turned on. A 3150 mL of an aqueous solution of **1a** (20 mM) and potassium carbonate (1.2 molar equiv.) was pumped (0.12 mL/min flow rate). The reaction was irradiated during 27 h. Samples were collected at selected times, concentrated under vacuum and analyzed by ^1^H NMR (D_2_O).

To isolate 6-allyl-6-azabicyclo[3.1.0]hex-3-en-2-ol (**2a**), 1360 mL of collected water corresponding to the period of 4 to 19 h of irradiation, was evaporated under vacuum and the obtained solid was dissolved in dichloromethane (3 × 250 mL), stirred for 15 min, and filtered. The solvent was evaporated to give 1.94g (52%) of **2a** as a brown oil.

*6-allyl-6-azabicyclo[3.1.0]hex-3-en-2-ol* (**2a**) ^1^H NMR (300 MHz, CDCl_3_) δ 6.32–6.29 (m, 1H), 5.95–5.86 (m, 2H), 5.23–5.19 (m, 2H), 4.12 (q, *J* = 7.1 Hz, 1H), 3.02–3.27 (m, 2H), 2.56–2.50 (m, 2H). Spectral data was in accordance with the literature [7].

## 4. Results

### 4.1. Batch Results

In order to achieve the photochemical transformation of **1a** to **2a** under continuous operation, we first established the best reaction conditions under batch. In this line, diverse parameters were evaluated (Table 1), such as reaction concentration (20, 40, 60, and 80 mM), residence time (up to 10 h), and the internal diameter of the QTs (0.2, 0.4 and 0.6 cm).

By using QT2, QT4 and QT6, with the same concentration (60 mM) of **1a** (Table 1—Entries 1, 2, 5), we observed that QT2, QT4 rapidly gave full conversion (2 and 4 h, respectively). This is due to the greater surface-to-volume ratio and to the shorter photoreactor diameter that circumvent nonuniform energy profiles [9]. However, for QT6 we obtained 82% conversion after 4 h of irradiation which means that the photon penetration in the solution is less effective. However, when looking for the productivity in mg h^−1^, the QT6 gave the best result (47.5 mg h^−1^ QT6 vs. 24.6 mg h^−1^ QT4 vs. 12.3 mg h^−1^ QT2; Table 1, entries 5, 2 and 1). This result is attributed to the larger internal diameter of QT6, allowing the production of **2a** in larger amount. We have chosen QT6 to continue our studies, based on the productivity results.

Since in a photoreaction the absorption of photons is correlated to the reaction concentration by the Bouguer−Lambert−Beer law [9,10,11], we then focused our attention on evaluating different concentrations of **1a** using QT6 (Table 1—Entries 3–6). As expected, the increase of concentration affected the photon penetration which led to the need of longer irradiation time to obtain a higher or full conversion. To our delight, we observed full conversion for 20 mM of **1a**, after 1 h of irradiation, and the best productivity result of 73.7 mg h^−1^ (Table 1—Entry 3). This was the best batch result and afforded the selected conditions to implement in the continuous-flow experiments.

### 4.2. Continuous-Flow Results

The batch studies shown QT6 as the selected tube for the continuous-flow experiments. Based on this, we set up the parallel quartz tube continuous-flow reactor using 12 quartz tubes assembled in the support (Figure S4 and Figure 2C). The new reactor was designated as PQT6: [12 tubes, 95 cm under irradiation (*l*) × 0.6 cm (*d*)], and has a surface-to-volume (S/V ratio) of 667 m^2^/m^3^ (Figure S4). With PQT6 reactor in hands, we started the flow experiments (Table 2—Entry 1), mimicking the best batch conditions (20 mM of **1a**, 1 h of residence time, Table 1—Entry 3).

**Figure 4:**
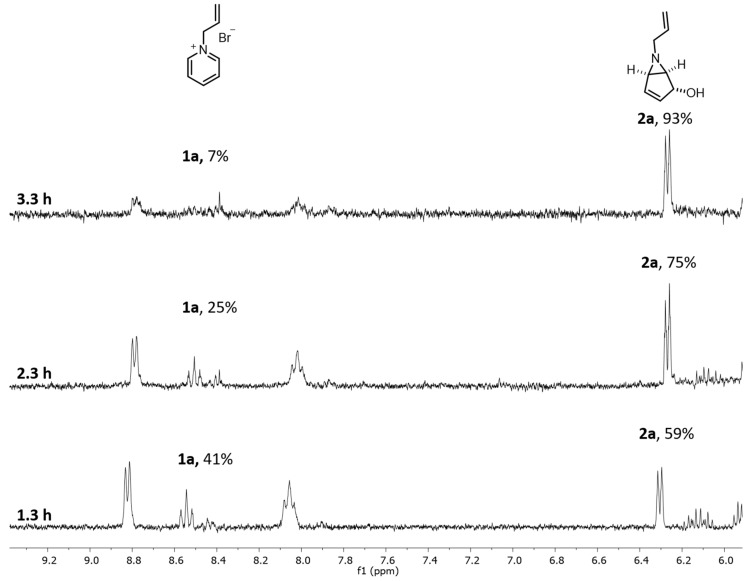
^1^H NMR profile for continuous-flow experiments optimization of **1a** to **2a** at 20 mM, with 1.3 h, 2.3 h and 3.3 h of residence time.

We programmed the peristaltic pump at 8.75 rpm (Table 2—Entry 1), which corresponds to a 0.35 mL/min flow rate and a 1.3 h residence time. However, after this time only 59% conversion was observed by ^1^H NMR analysis (Figure 4 and Table 2—Entry 1). The continuous process did not reproduce the batch experiment. Probably the 12 QTs of PTQ6 affected the photon flux in the system and consequently, less photons were available for the photochemical transformation. However, this situation is not new, as we observed the same behavior for the phototransformation of 1-butylpyridinium salt **1b** at 60 mM, under batch (FEP, S/V ratio = 1000 m^2^/m^3^; conversion—92%, irradiation time—8 h) and continuous-flow (FEP4 reactor, S/V ratio = 960 m^2^/m^3^, conversion—56%, irradiation time—8 h). We attributed this results to the geometry of FEP4, which could limit the photon flux [7].

In order to obtain a higher conversion of **1a** to **2a**, we decided to increase the residence time (Table 2—Entries 2, 3 and Figure 4). After 2.3 h (5 rpm, 0.21 mL/min) we obtained 75% conversion by ^1^H NMR (Figure 4) and for 3.3 h (3.5 rpm, 0.14 mL/min) a 93% conversion was achieved. With this result, we envisaged that 4 h of residence time would allow a full conversion of **1a** (0.12 mL/min flow rate; 3 rpm).

**Figure 5:**
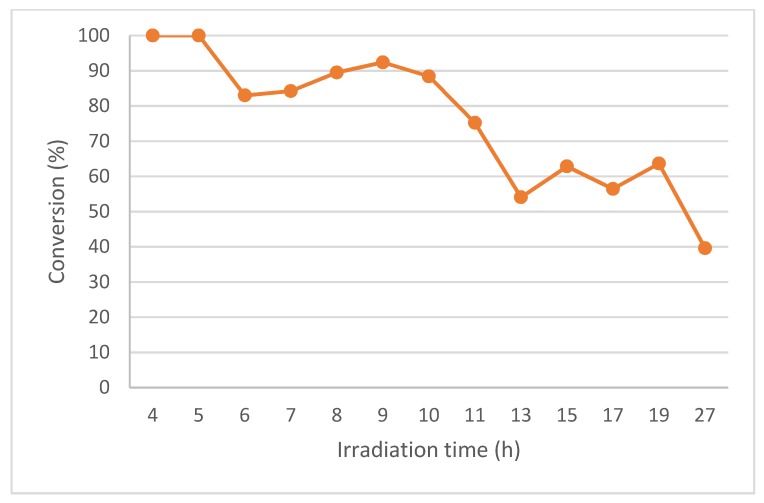
Conversion (%) for the continuous-photoflow of **1a** at 20 mM with 4 h of residence time using the PQT6 reactor.

We started the continuous-flow experience for process intensification of **2a**, *via* photoreaction of **1a** (Table 3) using 20 mM and 4 h of residence time (0.12 mL/min flow rate; 3 rpm). To start the experience, we filled the PQT6 with 350 mL of solution (Cycle 0) and then turned on the irradiation (Table 3, Entry 1). To our delight, the first collected samples with 4 h of residence time showed full conversion (Figure 5 and Table S8, Entries 2, 3). High conversions were observed on both Cycle 1, between 4–8 h of irradiation time (92%, Figure 5 and Table 3, Entry 2) and Cycle 2, between 8–13 h of irradiation time, (82%, Figure 5 and Table 3, Entry 3).

However, for Cycle 3 (13–19 h irradiation time), the conversion started to drop (66%, Figure 5 and Table 3, Entry 4) and continued to decrease for Cycle 4 (19–27 h irradiation time; 41%, Figure 5 and Table 3, Entry 5). This is probably due to the yellow polymer formed on the surface of the QTs during the irradiation (Figures S5C and S7) which reduce the photon flux. The formation of this yellow polymer was already observed previously by us [7].

For Cycle 1–3, which correspond to 15 h of irradiation time and 1.3 L of recovered volume, we isolated 1.94 g of **2a** (52% yield), allowing a space-time yield (STYs) of 129.3 mg h^−1^. This result is three times better than our previous one, obtained using PQT4 under recirculation continuous-flow condition, *e.g.*, STYs of 40.9 mg h^−1^ (1.2 g of isolated aziridine in 29.3 h of irradiation) [7]. In the same work, we only performed continuous-flow for FEP4 reactor and to prepare 6-butyl-6-azabicyclo[3.1.0]hex-3-en-2-ol, **2b**. This was due to the high dead volume of the PQT4 reactor, which limits its use under continuous operation, and also the difficulty of cleaning the FEP4 reactor caused by the formation of a yellow polymer during the irradiation time [7]. Herein, this is the first example of continuous-flow for the synthesis of **2a**.

## 5. Conclusions

We conducted the process intensification of **2a** under continuous-photoflow, using a home-made 12 parallel quartz tubes reactor (PTQ6) on a home-made UV reactor. The obtained space-time yield (129.3 mg h^−1^) improved 3 times when compared to our previous recirculating approach. In addition, we were able to prepare, for the first time under continuous flow, this valuable compound.

## Figures and Tables

**Table 1:** Selected results of photoreaction of **1a** to **2a** under batch ^1^.

Entry	Tube ^2^	S/V Ratio (m^2^/m^3^)	1a mM	Reaction Time (h)	Conv. (%) ^3^	Productivity (mg h^−1^)
1	QT2	2000	60	2	100	12.3
2	QT4	1000	60	4	100	24.6
3	QT6	667	20	1	100	73.7
4	QT6	667	40	6	100	24.6
5	QT6	667	60	4	82	47.5
6	QT6	667	80	8	77	28.4

^1^ All data for the batch reactions is available in the Supporting Information. ^2^ Quartz tubes with the same length and diameter of the ones used to assemble the continuous-flow reactor. ^3^ Determined by ^1^H NMR analysis.

**Table 2:** Optimization of photochemical transformation of **1a** to **2a** under continuous-flow.

Entry	Flow Rate mL/min	rpm	Residence Time (h)	Conv. (%) ^1^
1	0.35	8.75	1.3	59 ^2^
2	0.21	5	2.3	75 ^3^
3	0.14	3.5	3.3	93 ^3^

^1^ Determined by ^1^H NMR analysis. ^2^ Average results from 12 QTs under continuous-flow. ^3^ Result derived from analysis of 1 QT under continuous-flow, the remaining 11 QTs were under recirculating continuous-flow and were not considered for conversion analysis.

**Table 3:** Photochemical transformation of **1a** to **2a** under continuous-flow ^1^.

Entry	Reaction Time (h)	Cycle	Conv. (%) ^2^	Volume Out of the Reactor (mL)
1	0–4	0	49	350
2	4–8	1	92	360
3	8–13	2	82	450
4	13–19	3	66	550
5	19–27	4	41	710

^1^ Flow rate of 0.12 mL/min (3 rpm), residence time 4 h. ^2^ Determined by ^1^H NMR analysis.

## Supplementary Materials

The following data are available online at https://www.mdpi.com/2409-9279/2/3/67/s1: details of home-made UV reactor, home-made-continuous-flow reactors and obtained results, copies of ^1^H NMR spectra of photochemical crude reaction mixtures and synthesized compounds.
