# Supplementary material for: Process Intensification for the Synthesis of 6-Allyl-6-azabicyclo[3.1.0]hex-3-en-2-ol from 1-Allylpyridinium Salt Using a Continuous UV-Light Photoflow Approach
Source: Methods Protoc. 2019 Aug 5;2(3):67. doi: 10.3390/mps2030067 (PMC6789824; doi:10.3390/mps2030067)
Supplement: Supplementary file 1 [file mps-02-00067-s001.pdf]

Process intensification for the synthesis of 6-allyl-6-azabicyclo[3.1.0]hex-3-en-2-ol from 1-allylpyridinium salt using a continuous UV-light photoflow approach

Milene A. G. Fortunato[1], Chi-Phong Ly,[1,2] Filipa Siopa[1,*] and Carlos A. M. Afonso[1,*]

1 Research Institute for Medicines (iMed.ULisboa), Faculty of Pharmacy, Universidade de Lisboa, Av. Prof. Gama Pinto, 1649-003 Lisboa, Portugal.

2 Sorbonne Université, Faculté des Sciences et Ingénierie, CNRS, Institut Parisien de Chimie Moléculaire, IPCM, 4 place Jussieu, 75005 Paris, France.

* Correspondence: filipasiopa@ff.ulisboa.pt; carlosafonso@ff.ulisboa.pt

Supporting Information

Table of Contents

Pictures of home-made UV reactor and quartz tubes reactor used for this study 4

Figure S1: Home-made UV reactor containing 24 PURITEC HNS Germicidal lamps Ref. HNS 8W G5 G5 (8W at 254 nm) with 1,44 m length.; A) Top view of the reactor, 3 lamps assembled in series [8 x 3 = 24 lamps); B) Exterior view of the home-made UV reactor, with approximately 95 cm of irradiation zone; C) Interior view of half of the home-made UV reactor, is shown 4 blocks of lamps, in each one is shown 3 lamps in series 4

Figure S2: Quartz tubes' support for the home-made UV reactor B) Detailed view: Ring to attach the quartz tubes; C) Home-made continuous-flow parallel tube quartz reactor (PQT6): [12 tubes: 95 cm under irradiation (l) × 0.6 cm (d)] inside the home-made UV reactor (top view); D) Support with 3 tubes attached for batch experiments. 5

.... 5

Figure S3: Set up of the tubes for batch experiments; A) Quartz tubes' support for the home-made UV reactor with 3 tubes attached; B) Batch system inside the home-made UV reactor; C) Top view under irradiation. 5

Figure S4: Home-made continuous-flow parallel tube quartz reactor (PQT6) [12 tubes: 95 cm (l) × 0.6 cm (d)] A) Before UV irradiation, B) Top view of the PQT6 inside the home-made UV reactor, C) Top view of the PQT6 inside the home-made UV reactor under irradiation. 6

Figure S5: Home-made continuous-flow parallel tube quartz reactor (PQT6), [12 tubes: 95 cm (l) × 0.6 cm (d)] A) Top view before UV irradiation, B) top-view under irradiation; C) top-view after irradiation. 6

Figure S6: Equipment used for photochemical transformation in continuous flow 7

Troubleshooting..7

Figure S7: PQT6 after irradiation with yellow residue on the walls. 7

Batch experiments results..8

Batch experiments with different internal diameters (QT2, QT4, QT6).......................................8

Table S1: Results of the batch photochemical transformation of allyl pyridinium salt**,** Conc. **60 mM**, using QT2: [95 cm under irradiation (*l*) × 0.2 cm (*d*)]. (The results in green are also presented in Table 1 of the manuscript) 8

Figure S8: ^{1}H NMR spectra of the batch photochemical transformation of allyl pyridinium salt**,** Conc. **60 mM**, using a QT2, with 2 **hours** of irradiation time. 9

Table S2: Results of the batch photochemical transformation of allyl pyridinium salt**,** Conc. **60 mM**, using a QT4: [95 cm under irradiation (*l*) × 0.4 cm (*d*)]. (The results in green are also presented in Table 1 of the manuscript) 10

Figure S9: ^{1}H NMR spectra of the batch photochemical transformation of allyl pyridinium salt**,** Conc. **60 mM**, using a QT4, with 4 **hours** of irradiation time. 11

Figure S10: ^{1}H NMR spectra of the batch photochemical transformation of allyl pyridinium salt**,** Conc. **60 mM**, using a QT6, with 4 **hours** of irradiation time. 13

Batch studies different concentrations on the QT6 14

Table S4: Results of the batch photochemical transformation of allyl pyridinium salt, Conc. **20 mM**, using a QT6. (The results in green are also presented in Table 1 of the manuscript) 14

Figure S11: ^{1}H NMR spectra of the batch photochemical transformation of allyl pyridinium salt**,** Conc. **20 mM**, using a QT6, with **1 hour** of irradiation time. 15

Table S5: Results of the batch photochemical transformation of allyl pyridinium salt**,** Conc. **40 mM**, using a QT6. (The results in green are also presented in Table 1 of the manuscript) 16

Figure S12: ^{1}H NMR spectra of the batch photochemical transformation of allyl pyridinium salt, Conc. **40 mM**, using a QT6, with 6 hours of irradiation time. 17

Table S6: Results of the batch photochemical transformation of allyl pyridinium salt**,** Conc. **60 mM**, using a QT6 18

Figure S13: ^{1}H NMR spectra of the batch photochemical transformation of allyl pyridinium salt**,** Conc. **60 mM**, using a QT6, with **8 hours** of irradiation time. 19

Figure S14: ^{1}H NMR spectra of the batch photochemical transformation of allyl pyridinium salt**,** Conc. **80 mM**, using a QT6, with **8 hours** of irradiation time. 21

Figure S15: Comparison of the photoreaction of the allyl pyridinium salt, at Conc. 20, 40, 60, 80 mM using a QT6 [95 cm (l) × 0.6 cm (d)]: A) Conversion (%) and B) Productivity (mg/h) 22

Optimization of photochemical transformation of 1a to 2a under continuous-flow conditions . 23

Figure S16: ^{1}H NMR spectra of photochemical transformation of 1a to 2a under continuous-flow conditions [Flow rate: 0.35 mL/min; rpm: 8.75; Residence time: 1.3 h; Conversion: 59%] on the PTQ6 (Table 2, Entry 1). 23

Figure S17: ^{1}H NMR spectra of photochemical transformation of 1a to 2a under continuous-flow conditions [Flow rate: 0.21 mL/min; rpm: 5; Residence time: 2.3 h; Conversion: 75%] on the PTQ6 (Table 2, Entry 2). 24

Figure S18: ^{1}H NMR spectra of photochemical transformation of 1a to 2a under continuous-flow conditions [Flow rate: 0.14 mL/min; rpm: 3.5; Residence time: 3.3 h; Conversion: 93%] on the PTQ6 (Table 2, Entry 3). 25

Photochemical transformation of 1a to 2a under continuous-flow conditions. 25

Table S8: Photochemical transformation of 1a to 2a under continuous-flow conditions[1]. 25

Figure S20: Overview of combined ^{1}H NMR spectra of photochemical transformation of 1a to 2a under continuous-flow conditions [Flow rate: 0.12 mL/min; rpm: 3; Residence time: 4h] on the PTQ6 (Table S8, entries 2-14). 26

Figure S21: ^{1}H NMR spectra of photochemical transformation of 1a to 2a under continuous-flow conditions [Flow rate: 0.12 mL/min; rpm: 3; Residence time: 4h; Conversion: 92%] on the PTQ6 (Table 3 of the manuscript, Entry 2) 28

Figure S22: ^{1}H NMR spectra of photochemical transformation of 1a to 2a under continuous-flow conditions [Flow rate: 0.12 mL/min; rpm: 3; Residence time: 4h; Conversion: 83%] on the PTQ6 (Table 3 of the manuscript, Entry 2) 28

Figure S23: ^{1}H NMR spectra of photochemical transformation of 1a to 2a under continuous-flow conditions [Flow rate: 0.12 mL/min; rpm: 3; Residence time: 4h; Conversion: 66%] on the PTQ6 (Table 3 of the manuscript, Entry 4) 29

Figure S24: ^{1}H NMR spectra of photochemical transformation of 1a to 2a under continuous-flow conditions [Flow rate: 0.12 mL/min; rpm: 3; Residence time: 4h; Conversion: 41%] on the PTQ6 (Table 3 of the manuscript, Entry 5) 29

^{1}H NMR spectra 30

Figure S26: ^{1}H NMR spectra of 1-allylpyridinium bromide, in accordance with literature [7]. 30

Figure S27: ^{1}H NMR spectra of 6-allyl-6-azabicyclo[3.1.0]hex-3-en-2-ol, in accordance with literature [7]. 30

Pictures of home-made UV reactor and quartz tubes reactor used for this study

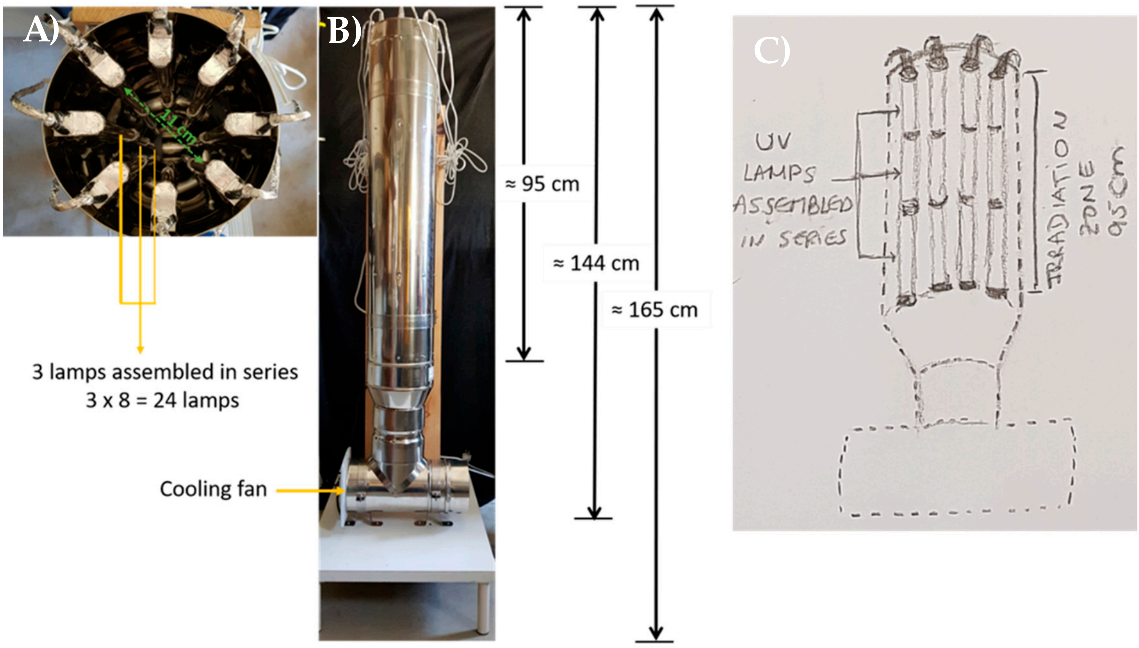

Figure S1: Home-made UV reactor containing 24 PURITEC HNS Germicidal lamps Ref. HNS 8W G5 G5 (8W at 254 nm) with 1,44 m length.; A) Top view of the reactor, 3 lamps assembled in series [8 x 3 = 24 lamps); B) Exterior view of the home-made UV reactor, with approximately 95 cm of irradiation zone; C) Interior view of half of the home-made UV reactor, is shown 4 blocks of lamps, in each one is shown 3 lamps in series .

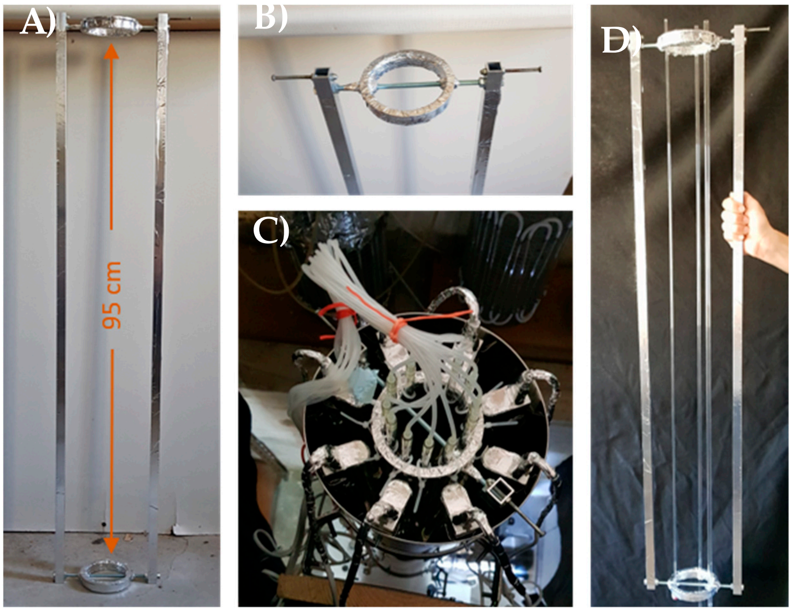

Figure S2: Quartz tubes' support for the home-made UV reactor B) Detailed view: Ring to attach the quartz tubes; C) Home-made continuous-flow parallel tube quartz reactor (PQT6): [12 tubes: 95 cm under irradiation (l) × 0.6 cm (d)] inside the home-made UV reactor (top view); D) Support with 3 tubes attached for batch experiments.

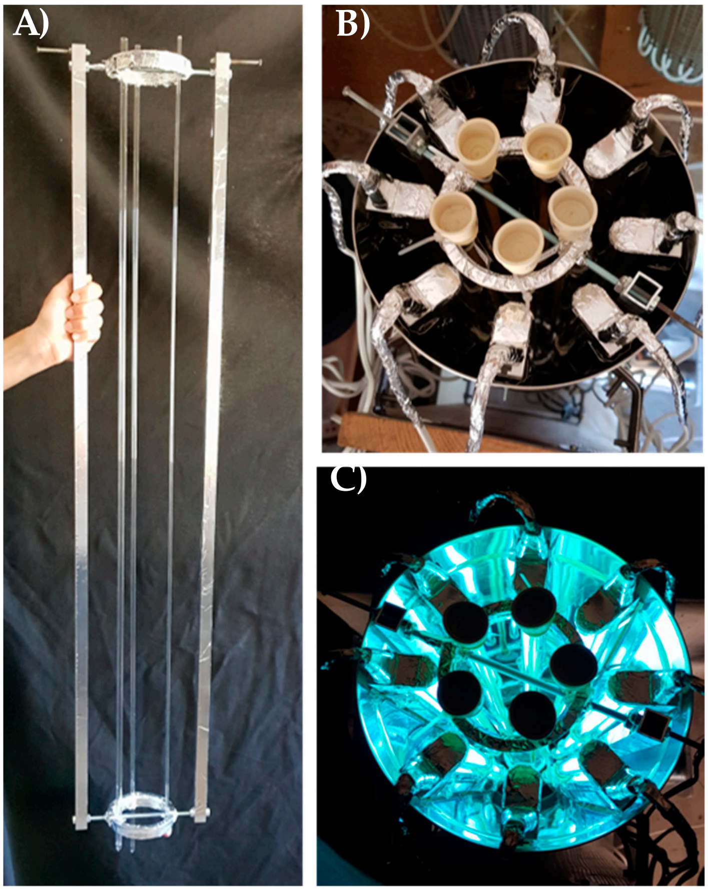

Figure S3: Set up of the tubes for batch experiments; A) Quartz tubes' support for the home-made UV reactor with 3 tubes attached; B) Batch system inside the home-made UV reactor; C) Top view under irradiation.

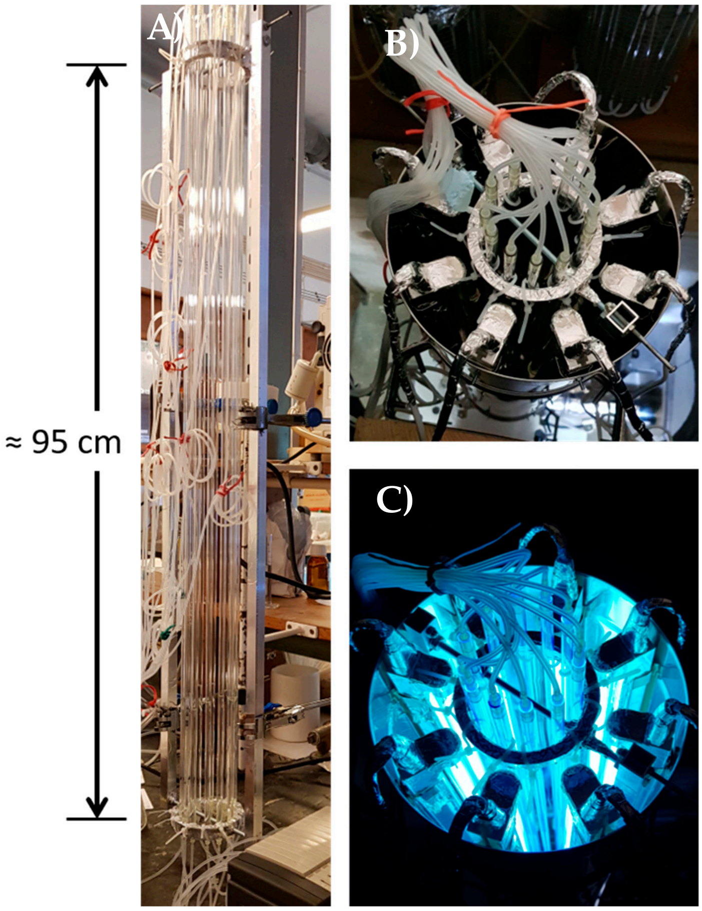

Figure S4: Home-made continuous-flow parallel tube quartz reactor (PQT6) [12 tubes: 95 cm (l) × 0.6 cm (d)] A) Before UV irradiation, B) Top view of the PQT6 inside the home-made UV reactor, C) Top view of the PQT6 inside the home-made UV reactor under irradiation.

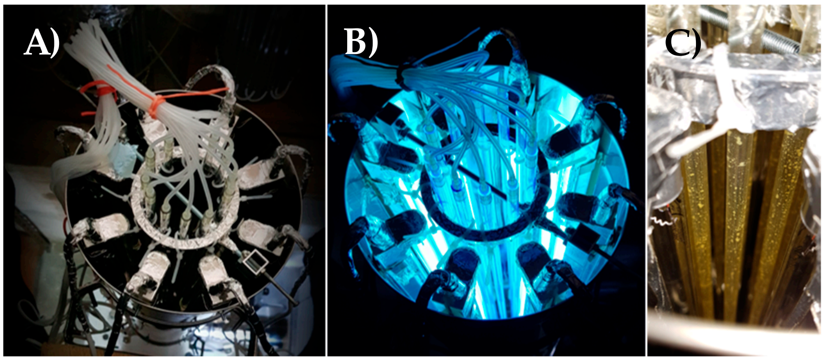

Figure S5: Home-made continuous-flow parallel tube quartz reactor (PQT6), [12 tubes: 95 cm (l) × 0.6 cm (d)] A) Top view before UV irradiation, B) top-view under irradiation; C) top-view after irradiation.

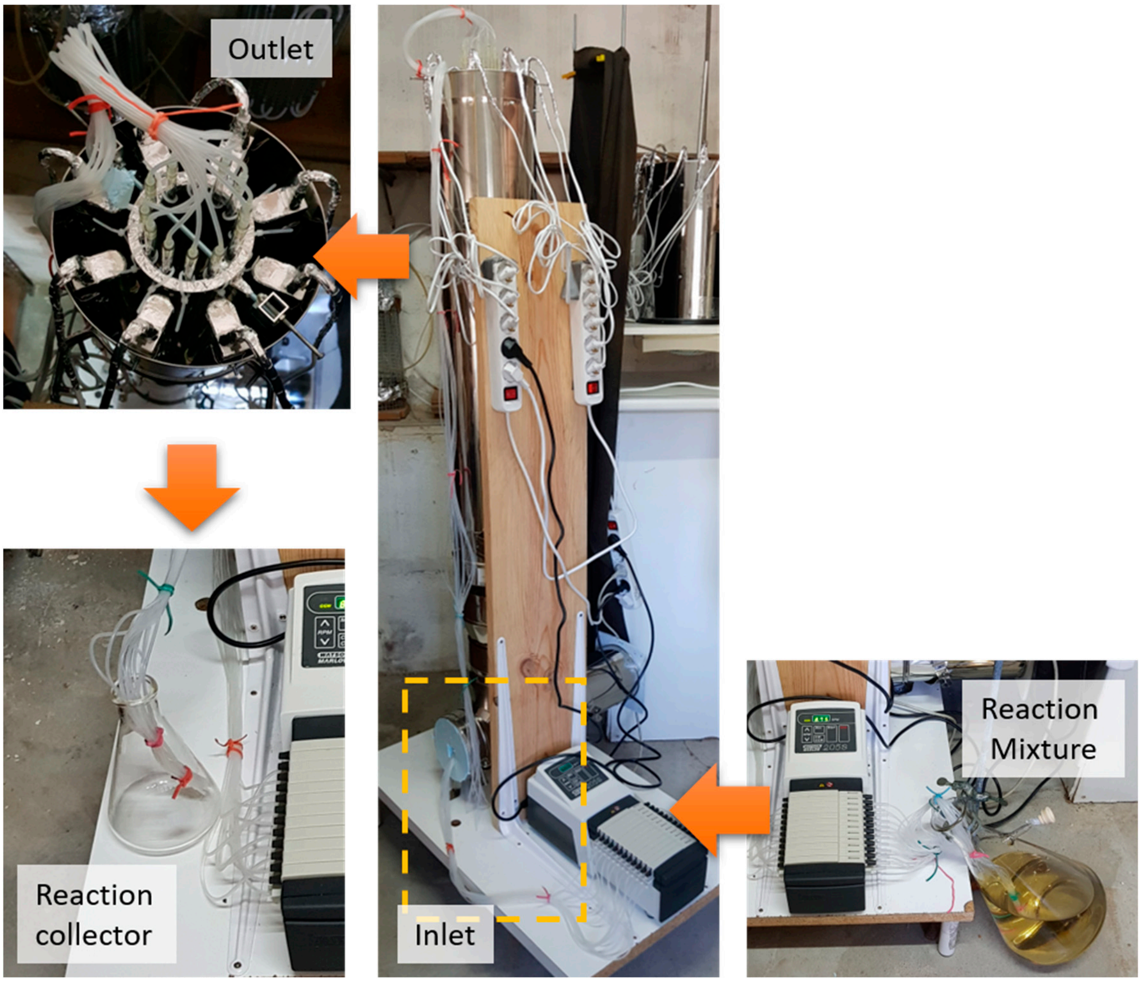

Figure S6: Equipment used for photochemical transformation in continuous flow

Troubleshooting

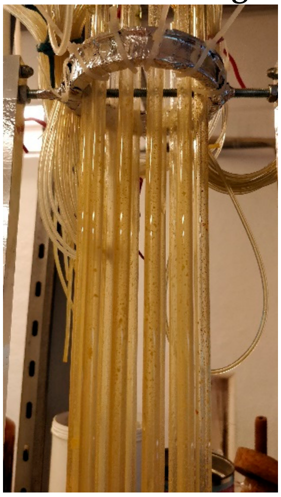

Figure S7: PQT6 after irradiation with yellow residue on the walls.

In all the photochemical reactions a yellow residue was gradually formed on the reactor walls. The residue was removed by disassembling the PQT6 and washing the quartz tubes using water pressure and a tube cleaning brush.

Batch experiments results

Batch experiments with different internal diameters (QT2, QT4, QT6)

Table S1: Results of the batch photochemical transformation of allyl pyridinium salt**,** Conc. **60 mM**, using QT2: [95 cm under irradiation (*l*) × 0.2 cm (*d*)]. (The results in green are also presented in Table 1 of the manuscript)

Time (h)	Conv.(%)[1]	Conv.(%)[2]	Avg. Conv. (%)[3]	Product Mass (g)[4]	Productivity g[4] L^{-1} [5] h^{-1}	Productivity g[4] m^{-2} [6] h^{-1}	Productivity mg h^{-1}
1	80.97	81.63	81.30	0.020	6.69	3.35	19.97
2	100	100	100	0.0246	4.12	2.06	12.28
3	100	100	100	0.0246	2.74	1.37	8.19
4	100	100	100	0.0246	2.06	1.03	6.14
5	100	100	100	0.0246	1.65	0.82	4.91

[1] ^{1}H NMR conversion obtained by the integration of the aziridine peak at 6.30 ppm to 1H and the pyridine peak at 8.81 ppm. $Conversion = \frac{\text{Area of Aziridine signal (6.30 ppm)}}{\text{[Area of Pyridine signal (8.81 ppm)/2+ Area of Aziridine signals (6.30 ppm)]}} x100$

[2] ^{1}H NMR conversion obtained by the integration of the aziridine peak at 6.30 pm to 1H and the pyridine peak at 8.05 ppm. $Conversion = \frac{\text{Area of Aziridine signal (6.30 ppm)}}{\text{[Area of Pyridine signal (8.05 ppm)/2+ Area of Aziridine signals (6.30 ppm)]}} x100$

[3] Average of the Conversions

[4] $Product\ mass\ (g) = \frac{\text{[Avg.Conversion (\%)*MM Aziridine (138.17 g/mol)*n mol A1ziridine (0.000179 mol)]}}{100}$

[5] Irradiation Volume - $\pi \times r^2 \times h = \pi \times 0.1^2 \times 95 = 2.99\ cm^3$ = 0.00299L

[6] Area = $L \times 2\pi r = 0.95 \times 2\pi \times 0.001 = 0.006\ m^2$

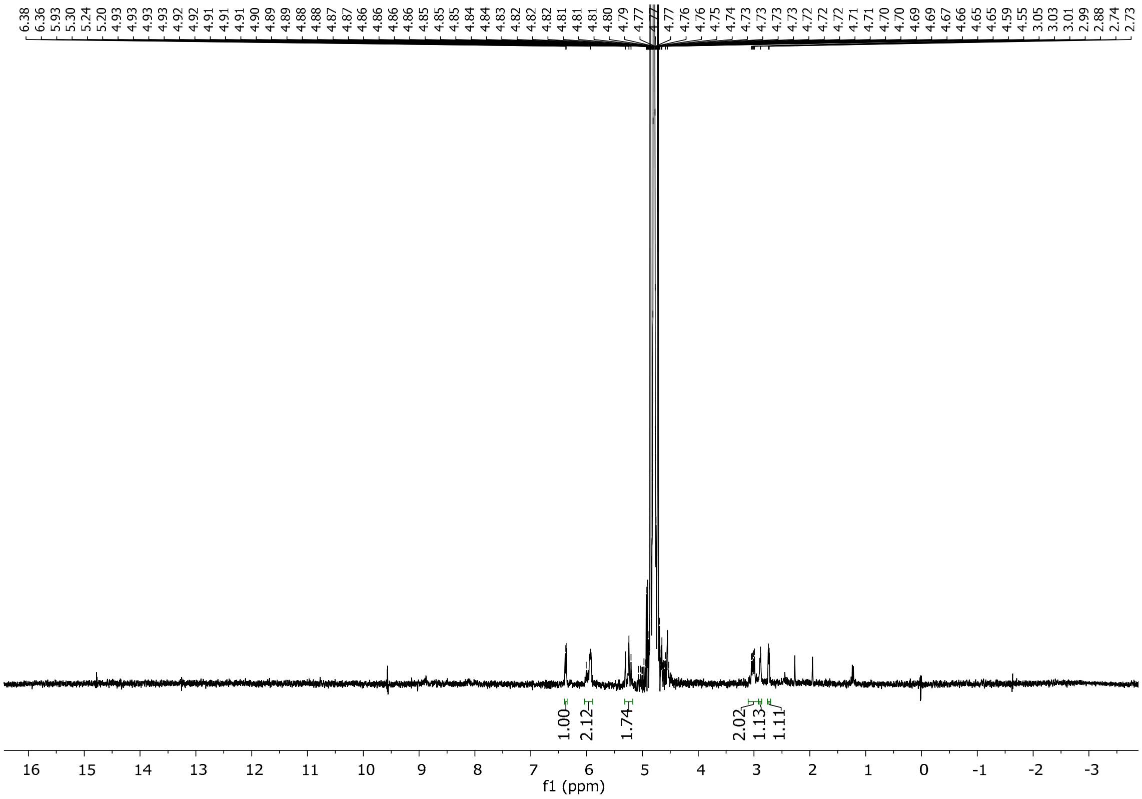

Figure S8: ^{1}H NMR spectra of the batch photochemical transformation of allyl pyridinium salt**,** Conc. **60 mM**, using a QT2, with 2 **hours** of irradiation time.

Table S2: Results of the batch photochemical transformation of allyl pyridinium salt**,** Conc. **60 mM**, using a QT4: [95 cm under irradiation (*l*) × 0.4 cm (*d*)]. (The results in green are also presented in Table 1 of the manuscript)

Time (h)	Conv.(%)[1]	Conv.(%)[2]	Avg. Conv. (%)[3]	Product Mass (g)[4]	Productivity $g^{[4]}$ L^{-1} [5] h^{-1}	Productivity $g^{[4]}$ m^{-2} [6] h^{-1}	Productivity mg h^{-1}
2	80.97	79.37	80.17	0.079	3.30	3.30	39.38
4	90.50	87.34	88.92	0.087	1.83	1.83	21.84
5	90.50	88.89	89.69	0.088	1.48	1.48	17.63
6	86.96	84.75	85.85	0.084	1.18	1.18	14.06
7	92.59	9132	91.96	0.090	1.08	1.08	12.91
8	92.17	92.17	92.17	0.091	0.95	0.95	11.32

[1] ^{1}H NMR conversion obtained by the integration of the aziridine peak at 6.30 ppm to 1H and the pyridine peak at 8.81 ppm. $Conversion = \frac{\text{Area of Aziridine signal (6.30 ppm)}}{[\text{Area of Pyridine signal (8.81 ppm)/2+ Area of Aziridine signals (6.30 ppm)}]} \text{x100}$

[2] ^{1}H NMR conversion obtained by the integration of the aziridine peak at 6.30 pm to 1H and the pyridine peak at 8.05 ppm. $Conversion = \frac{\text{Area of Aziridine signal (6.30 ppm)}}{[\text{Area of Pyridine signal (8.05 ppm)/2+ Area of Aziridine signals (6.30 ppm)}]} \text{x100}$

[3] Average of the Conversions

[4] $Product\ mass\ (g) = \frac{[\text{Avg.Conversion (\%)} * \text{MM Aziridine (137.18g/mol)} * \text{n mol Aziridine (0.000716mol)}]}{100}$

[5] Irradiation Volume- $\pi \times r^2 \times h = \pi \times 0.2^2 \times 95 = 11.94\ cm^3 = 0.01194L$

[6] Area = $L \times 2\pi\ r = 0.95 \times 2\pi \times 0.002 = 0.01194\ m^2$

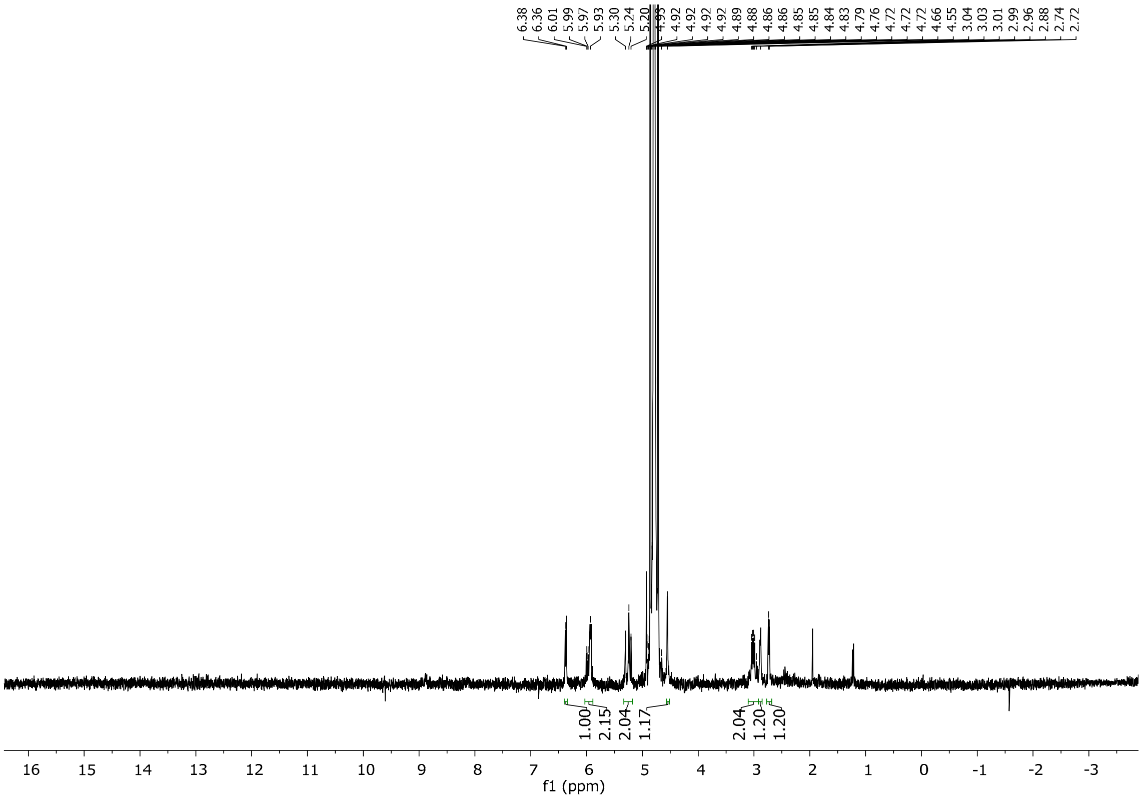

Figure S9: ^{1}H NMR spectra of the batch photochemical transformation of allyl pyridinium salt**,** Conc. **60 mM**, using a QT4, with 4 **hours** of irradiation time.

Table S3: Results of the batch photochemical transformation of allyl pyridinium salt**,** Conc. **60 mM**, using a QT6: [95 cm under irradiation (*l*) × 0.6 cm (*d*)]. (The results in green are also presented in Table 1 of the manuscript)

**Time (h)**	**Conv.(%)[1]**	**Conv.(%)[2]**	**Avg. Conv. (%)[3]**	**Product Mass (g)[4]**	**Productivity g[4] L^{-1} [5] h^{-1}**	**Productivity g[4] m^{-2} [6] h^{-1}**	**Productivity mg h^{-1}**
2	65.36	63.90	64.63	0.14	2.64	3.97	71.04
4	86.96	84.75	85.85	0.19	1.77	2.65	47.53
6	94.79	94.34	94.56	0.21	1.30	1.95	35.01
8	91.74	100	95.87	0.21	0.99	1.48	26.53
10	93.90	4.34	94.12	0.21	0.77	1.16	20.78

[1] ^{1}H NMR conversion obtained by the integration of the aziridine peak at 6.30 ppm to 1H and the pyridine peak at 8.81 ppm. $Conversion = \frac{\text{Area of Aziridine signal (6.30 ppm)}}{\text{[Area of Pyridine signal (8.81 ppm)/2+ Area of Aziridine signals (6.30 ppm)]}} x100$

[2] ^{1}H NMR conversion obtained by the integration of the aziridine peak at 6.30 pm to 1H and the pyridine peak at 8.05 ppm. $Conversion = \frac{\text{Area of Aziridine signal (6.30 ppm)}}{\text{[Area of Pyridine signal (8.05 ppm)/2+ Area of Aziridine signals (6.30 ppm)]}} x100$

[3] Average of the Conversions

[4] $Product\ mass\ (g) = \frac{\text{[Avg.Conversion (\%)*MM Aziridine (138.17 g/mol)*n mol Aziridine (0.00161 mol)]}}{100}$

[5] Irradiation Volume- $\pi \times r^2 \times h = \pi \times 0.3^2 \times 95 = 26.86\ cm^3 = 0.0269L$

[6] Area = $L \times 2\pi r = 0.95 \times 2\pi \times 0.003 = 0.0179\ m^2$

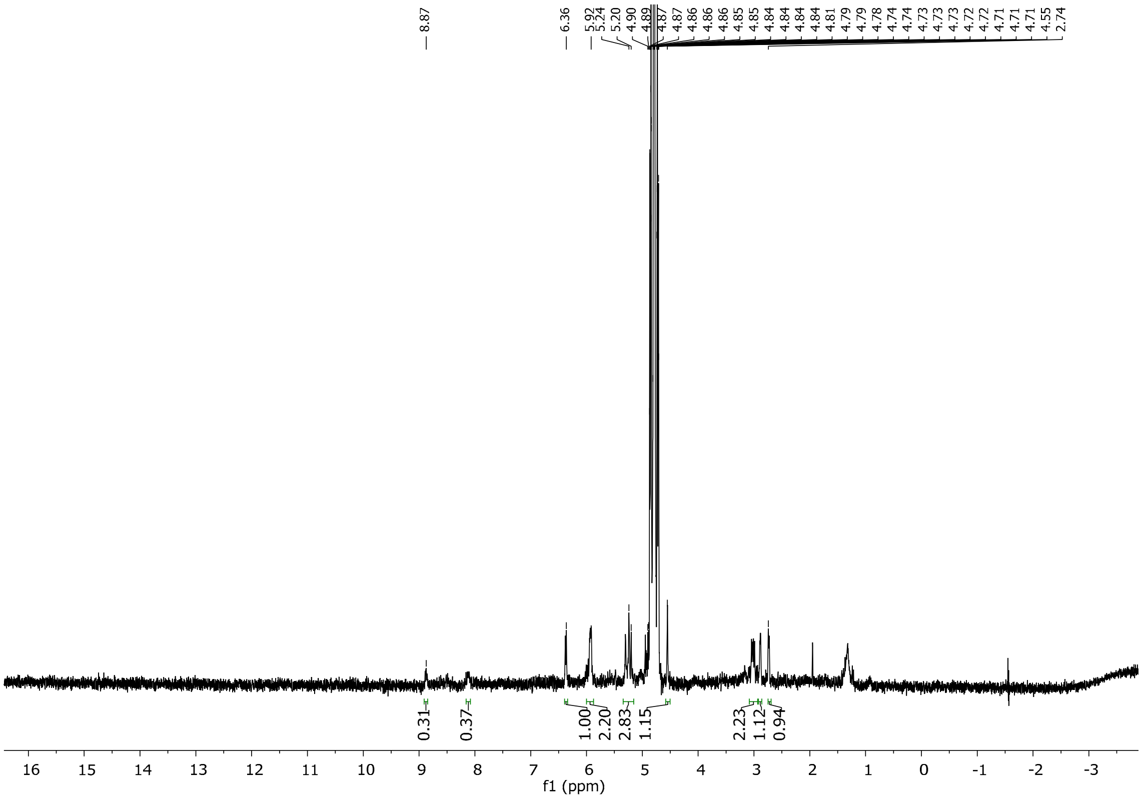

Figure S10: ^{1}H NMR spectra of the batch photochemical transformation of allyl pyridinium salt**,** Conc. **60 mM**, using a QT6, with 4 **hours** of irradiation time.

Batch studies different concentrations on the QT6

Table S4: Results of the batch photochemical transformation of allyl pyridinium salt, Conc. **20 mM**, using a QT6. (The results in green are also presented in Table 1 of the manuscript)

Time (h)	Conv.(%)[1]	Conv.(%)[2]	Avg. Conv. (%)[3]	Product Mass (g)[4]	Productivity $g^{[4]}\ L^{-1\,[5]}\ h^{-1}$	Productivity $g^{[4]}\ m^{-2\,[6]}\ h^{-1}$	Productivity $mg\ h^{-1}$
1	100	100	100	0.074	2.74	4.12	73.69
2	100	100	100	0.074	1.37	2.06	36.85
3	100	100	100	0.074	0.91	1.37	24.56
4	100	100	100	0.074	0.69	1.03	18.42
5	100	100	100	0.074	0.55	0.82	14.74
6	100	100	100	0.074	0.46	0.69	12.28
7	100	100	100	0.074	0.39	0.59	10.53

[1] ^{1}H NMR conversion obtained by the integration of the aziridine peak at 6.30 ppm to 1H and the pyridine peak at 8.81 ppm. $Conversion = \frac{\text{Area of Aziridine signal (6.30 ppm)}}{[\text{Area of Pyridine signal (8.81 ppm)/2+ Area of Aziridine signals (6.30 ppm)}]} \text{x}100$

[2] ^{1}H NMR conversion obtained by the integration of the aziridine peak at 6.30 pm to 1H and the pyridine peak at 8.05 ppm. $Conversion = \frac{\text{Area of Aziridine signal (6.30 ppm)}}{[\text{Area of Pyridine signal (8.05 ppm)/2+ Area of Aziridine signals (6.30 ppm)}]} \text{x}100$

[3] Average of the Conversions

[4] $Product\ mass\ (g) = \frac{[\text{Avg.Conversion (\%)}*\text{MM Aziridine (138.17 g/mol)}*\text{n mol Aziridine (0.000537 mol)}]}{100}$

[5] Irradiation Volume- $\pi \times r^2 \times h = \pi \times 0.3^2 \times 95 = 26.86\ cm^3 = 0.0269L$

[6] Area = $L \times 2\pi\ r = 0.95 \times 2\pi \times 0.003 = 0.0179\ m^2$

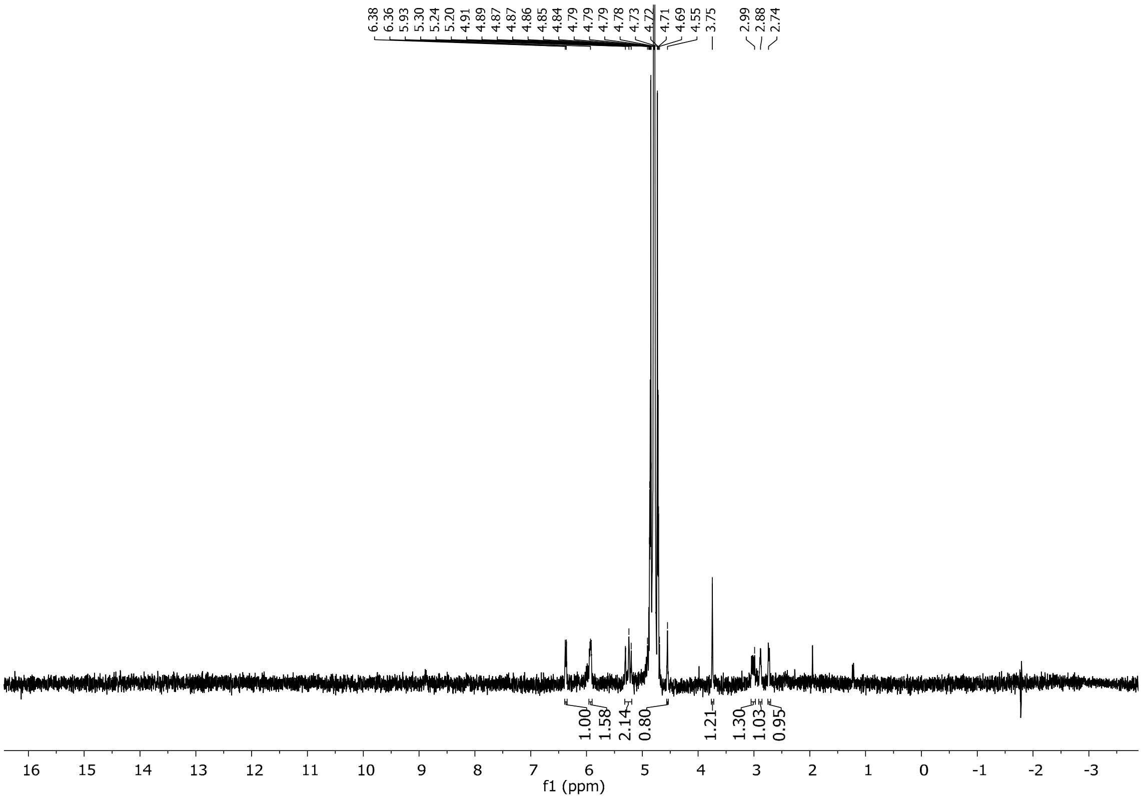

Figure S11: ^{1}H NMR spectra of the batch photochemical transformation of allyl pyridinium salt**,** Conc. **20 mM**, using a QT6, with **1 hour** of irradiation time.

Table S5: Results of the batch photochemical transformation of allyl pyridinium salt**,** Conc. **40 mM**, using a QT6. (The results in green are also presented in Table 1 of the manuscript)

Time (h)	Conv.(%)[1]	Conv.(%)[2]	Avg. Conv. (%)[3]	Product Mass (g)[4]	Productivity g[4] L^{-1} [5] h^{-1}	Productivity g[4] m^{-2} [6] h^{-1}	Productivity mg h^{-1}
1	60.8	57.3	59.05	0.087	3.24	4.86	87.03
2	78.13	76.34	77.23	0.113	2.11	3.17	56.74
3	83.33	86.21	84.77	0.125	1.55	2.33	41.76
4	86.96	88.50	87.73	0.130	1.21	1.81	32.43
5	93.46	92.59	93.03	0.137	1.02	1.53	27.41
6	100	100	100	0.7	0.915	1.37	24.56

[4] $Product\ mass\ (g) = \frac{[\text{Avg.Conversion (\%)} * \text{MM Aziridine (138.17 g/mol)} * \text{n mol Aziridine (0.00107 mol)}]}{100}$

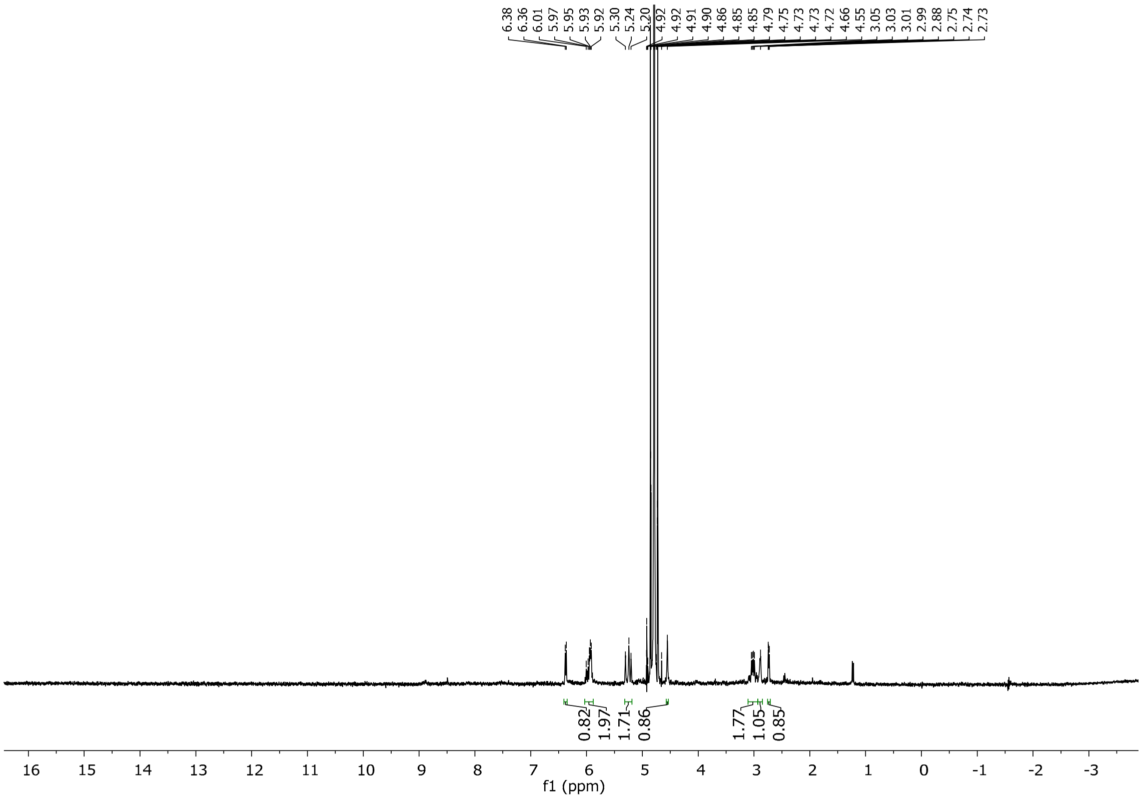

Figure S12: ^{1}H NMR spectra of the batch photochemical transformation of allyl pyridinium salt, Conc. **40 mM**, using a QT6, with 6 hours of irradiation time.

Table S6: Results of the batch photochemical transformation of allyl pyridinium salt**,** Conc. **60 mM**, using a QT6

Time (h)	Conv.(%)[1]	Conv.(%)[2]	Avg. Conv. (%)[3]	Product Mass (g)[4]	Productivity g[4] L^{-1} [5] h^{-1}	Productivity g[4] m^{-2} [6] h^{-1}	Productivity mg h^{-1}
2	61.16	59.70	60.43	0.133	2.47	3.70	66.33
4	78.43	77.52	77.98	0.172	1.61	2.41	43.11
6	86.21	84.75	85.48	0.190	1.18	1.77	31.69
8	87.72	90.09	88.90	0.197	0.9156	1.37	24.60

[4] $Product\ mass\ (g) = \frac{[\text{Avg.Conversion (\%)} * \text{MM Aziridine (138.17 g/mol)} * \text{n mol Aziridine (0.00161 mol)}]}{100}$

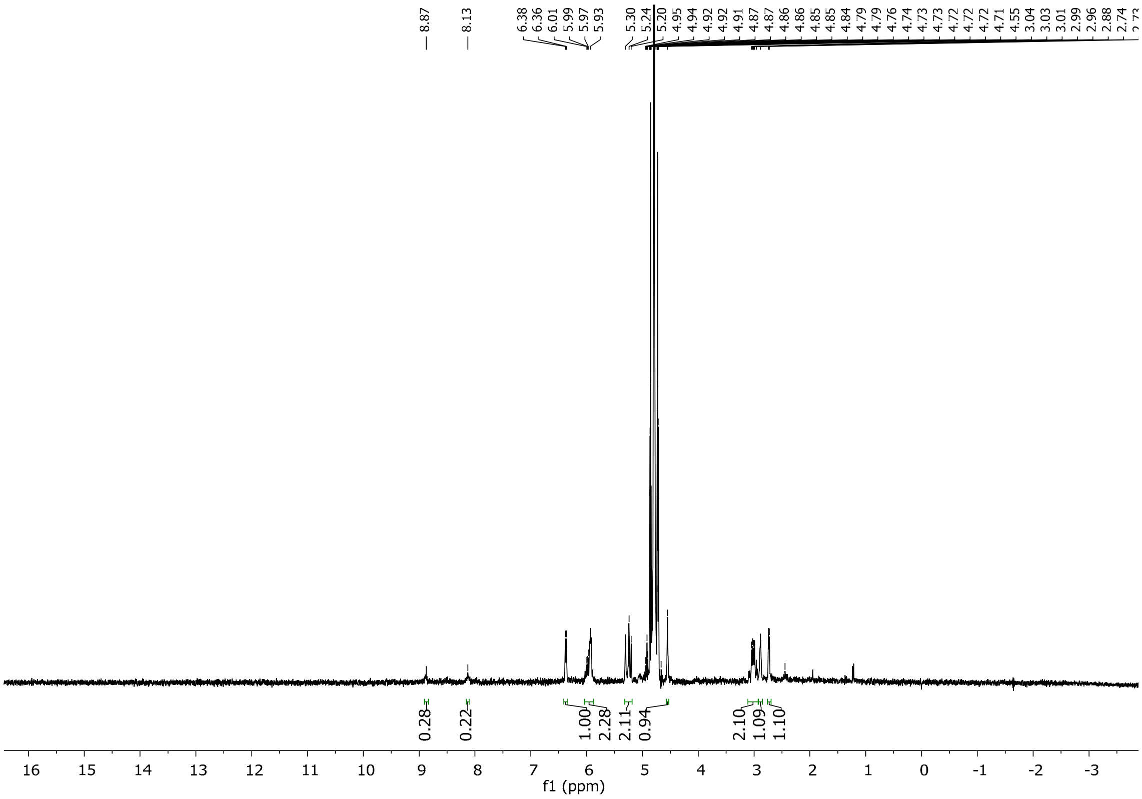

Figure S13: ^{1}H NMR spectra of the batch photochemical transformation of allyl pyridinium salt**,** Conc. **60 mM**, using a QT6, with **8 hours** of irradiation time.

Table S7: Results of the batch photochemical transformation of allyl pyridinium salt**,** Conc. **80 mM**, using a QT6. (The results in green are also presented in Table 1 of the manuscript)

Time (h)	Conv.(%)[1]	Conv.(%)[2]	Avg. Conv. (%)[3]	Product Mass (g)[4]	Productivity g[4] L^{-1} [5] h^{-1}	Productivity g[4] m^{-2} [6] h^{-1}	Productivity mg h^{-1}
2	52.77	59.70	60.43	0.133	2.47	3.70	76.64
4	60.98	77.52	77.98	0.172	1.61	2.41	44.22
6	68.73	67.80	68.26	0.200	1.24	1.87	33.41
8	78.13	76.34	77.23	0.227	1.06	1.58	28.37

[4] $Product\ mass\ (g) = \frac{[\text{Avg.Conversion (\%)}*\text{MM Aziridine (138.17 g/mol)}*\text{n mol Aziridine (0.00215 mol)}]}{100}$

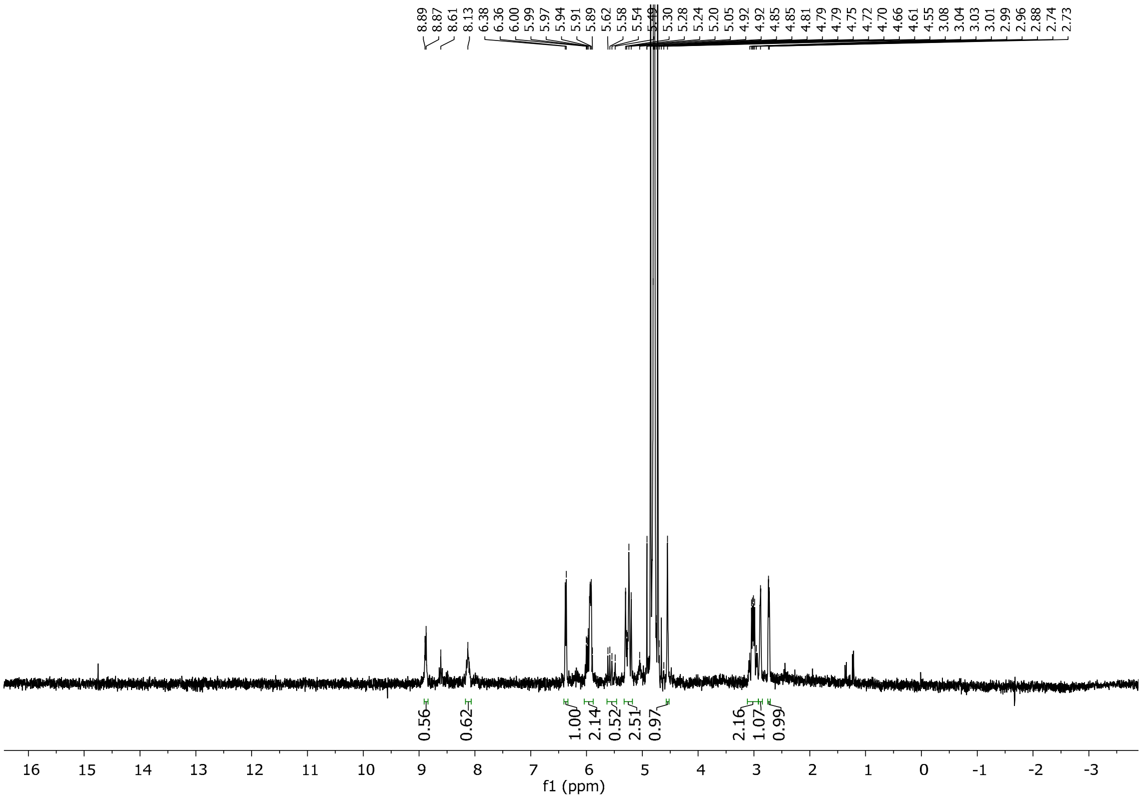

Figure S14: ^{1}H NMR spectra of the batch photochemical transformation of allyl pyridinium salt**,** Conc. **80 mM**, using a QT6, with **8 hours** of irradiation time.

A)

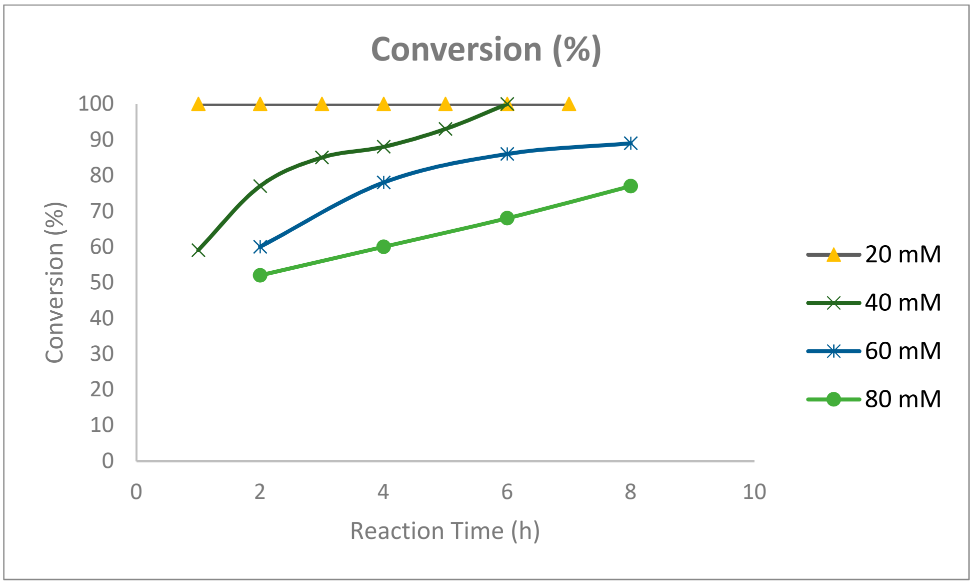

B)

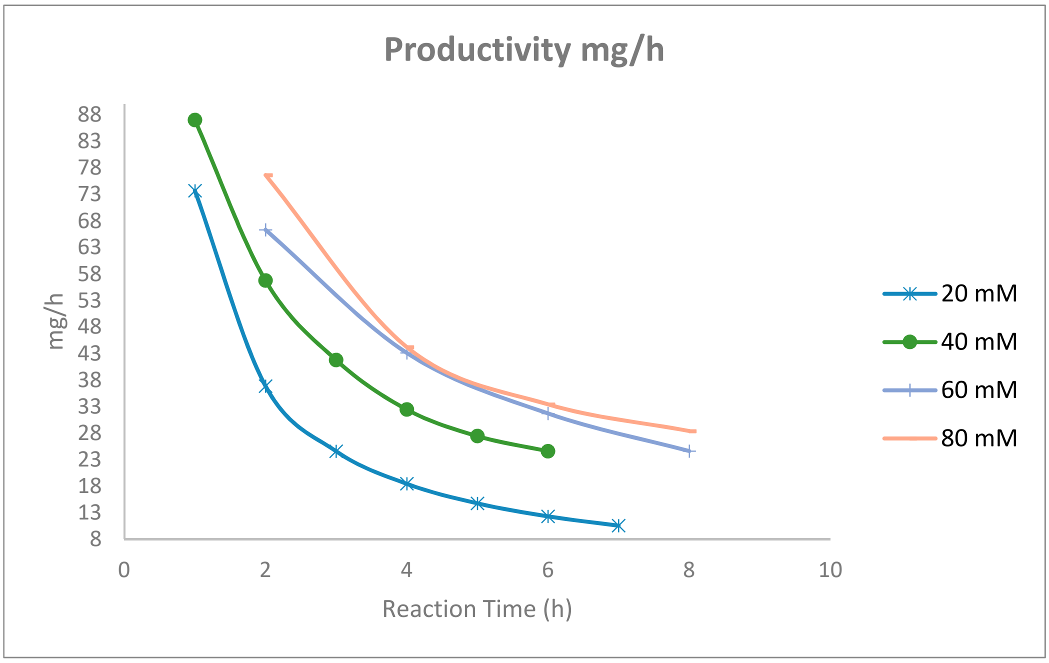

Figure S15: Comparison of the photoreaction of the allyl pyridinium salt, at Conc. 20, 40, 60, 80 mM using a QT6 [95 cm (l) × 0.6 cm (d)]: A) Conversion (%) and B) Productivity (mg/h).

Optimization of photochemical transformation of 1a to 2a under continuous-flow conditions

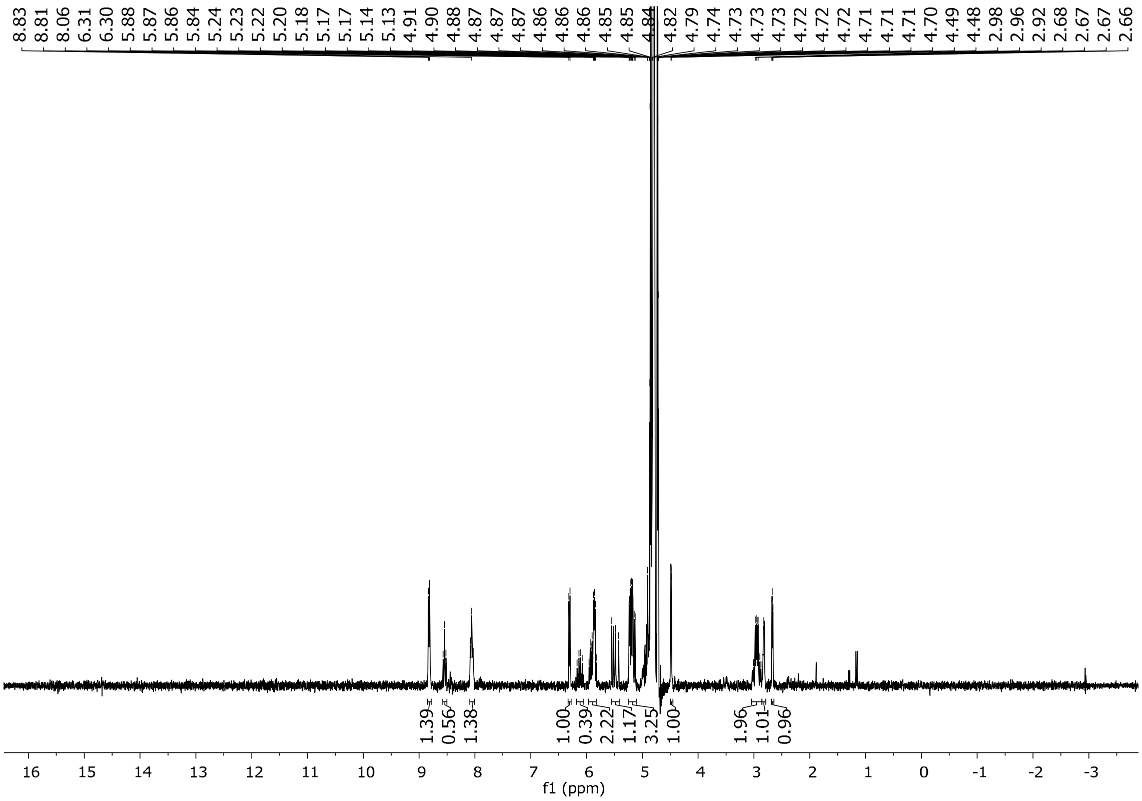

Figure S16: ^{1}H NMR spectra of photochemical transformation of 1a to 2a under continuous-flow conditions [Flow rate: 0.35 mL/min; rpm: 8.75; Residence time: 1.3 h; Conversion: 59%] on the PTQ6 (Table 2, Entry 1).

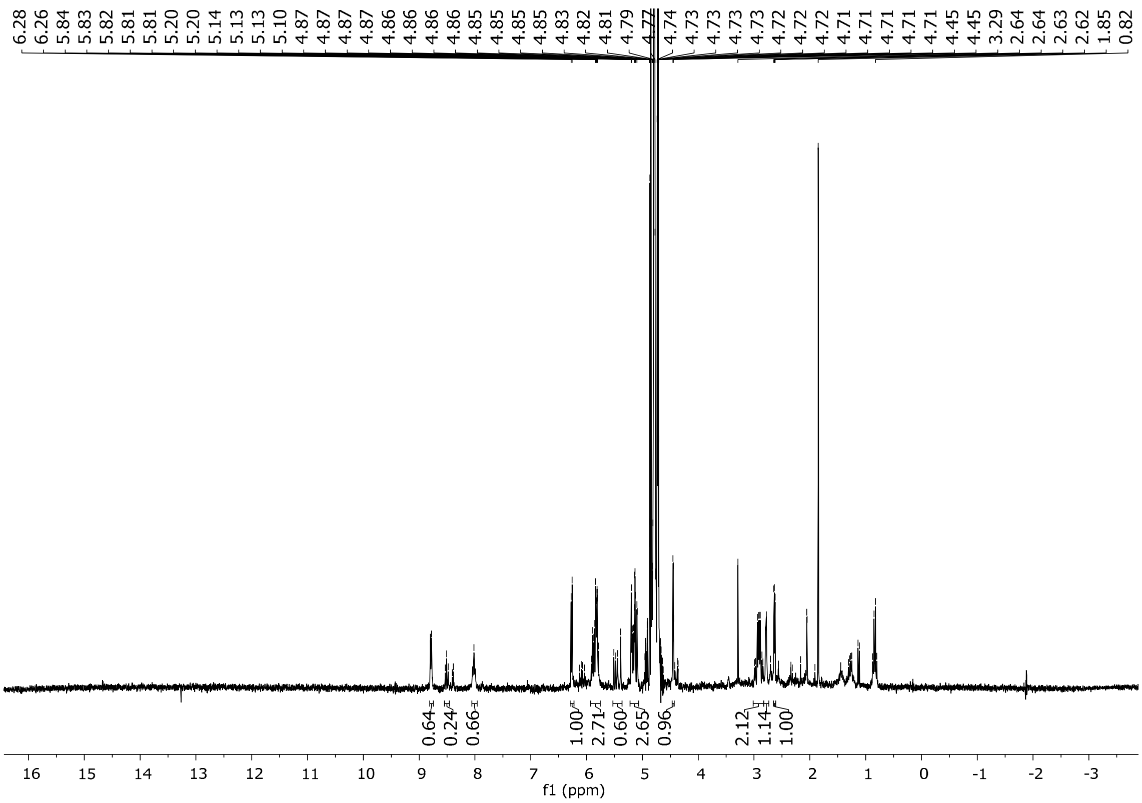

Figure S17: ^1H NMR spectra of photochemical transformation of 1a to 2a under continuous-flow conditions [Flow rate: 0.21 mL/min; rpm: 5; Residence time: 2.3 h; Conversion: 75%] on the PTQ6 (Table 2, Entry 2).

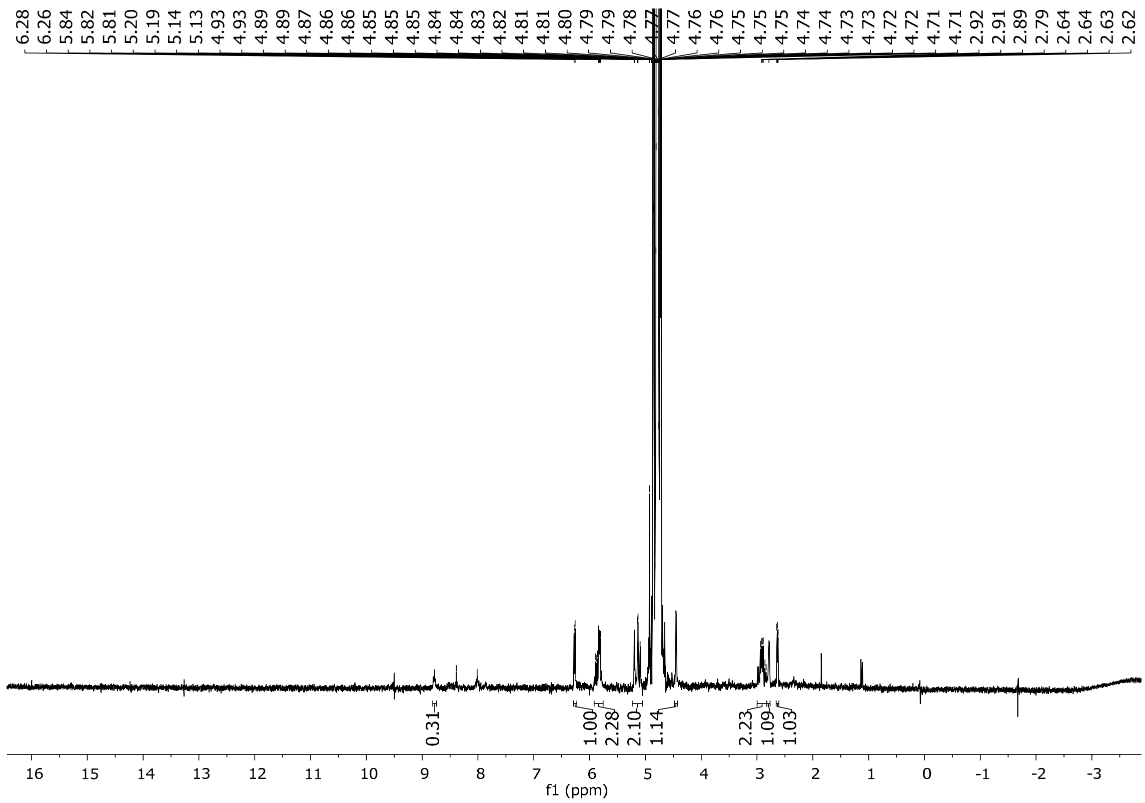

Figure S18: ^{1}H NMR spectra of photochemical transformation of 1a to 2a under continuous-flow conditions [Flow rate: 0.14 mL/min; rpm: 3.5; Residence time: 3.3 h; Conversion: 93%] on the PTQ6 (Table 2, Entry 3).

Photochemical transformation of 1a to 2a under continuous-flow conditions.

Table S8: Photochemical transformation of 1a to 2a under continuous-flow conditions[1].

Entry	Irradiation time (h)	Fraction	Conv. (%)[2]	Cycle	Volume out of the reactor (mL)
1	0 - 4	0	49	0	350
2	4	F1	100	-	-
3	5	F2	100[3]	-	-
4	6	F3	83	-	-
5	7	F4	84	-	-
6	8	F5	89	1	360
7	9	F6	92	-	-
8	10	F7	88	-	-
9	11	F8	75	-	-
10	13	F9	54	2	450
11	15	F10	63	-	-
12	17	F11	56	-	-
13	19	F12	64	3	550
14	27	F13	40	4	710

[1] Flow rate of 0.12 mL/min (3 rpm), residence time 4 h. [2] Determined by ^{1}H NMR. [3]Data not showed

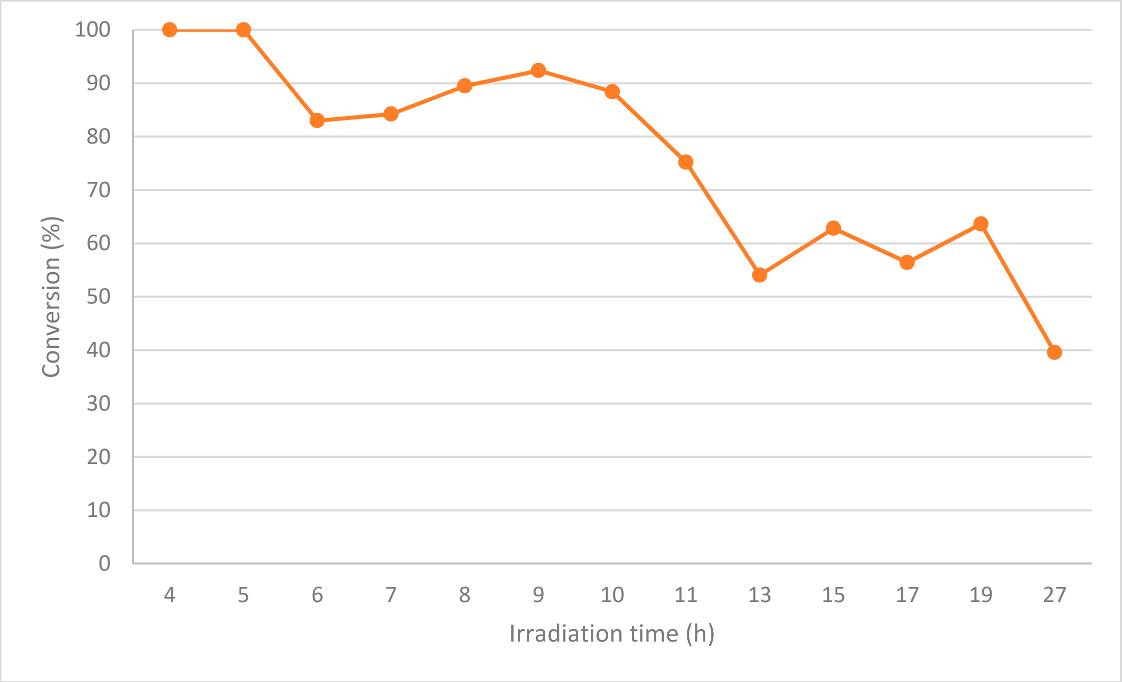

Figure S19: Conversion (%) for the continuous-photoflow of **1a** at 20 mM with 4 h of residence time using the PQT6 reactor.

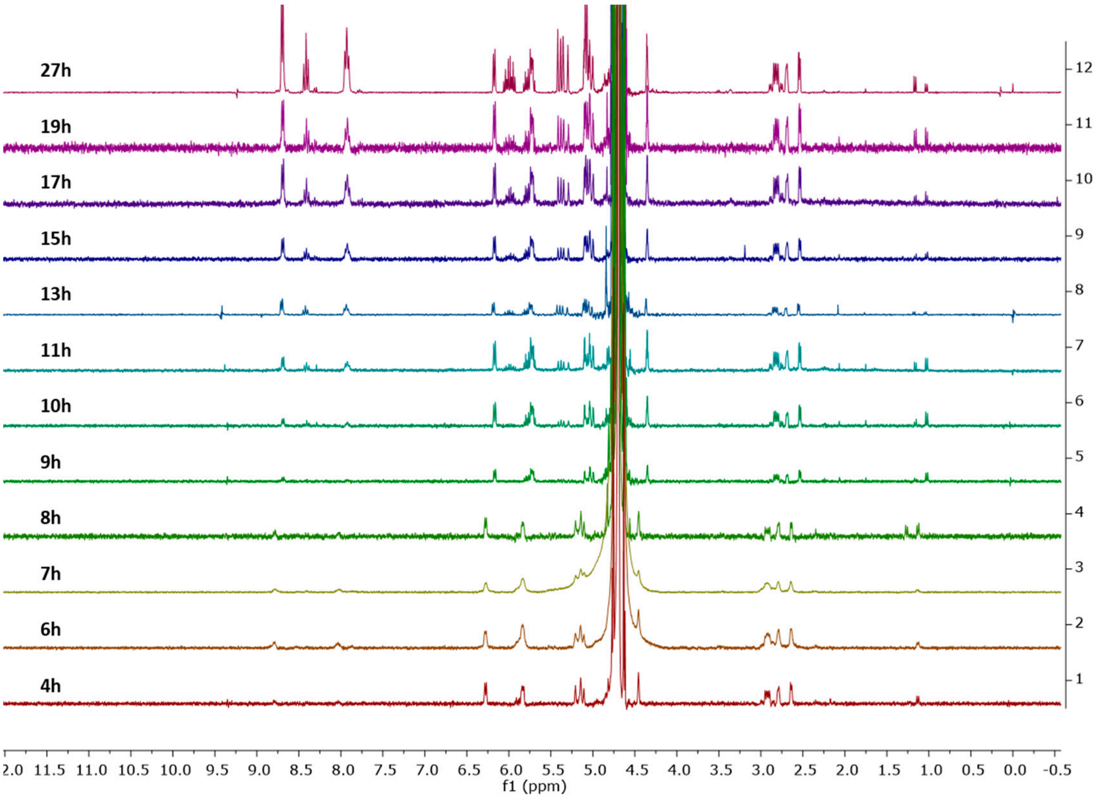

Figure S20: Overview of combined ^{1}H NMR spectra of photochemical transformation of 1a to 2a under continuous-flow conditions [Flow rate: 0.12 mL/min; rpm: 3; Residence time: 4h] on the PTQ6 (Table S8, entries 2-14).

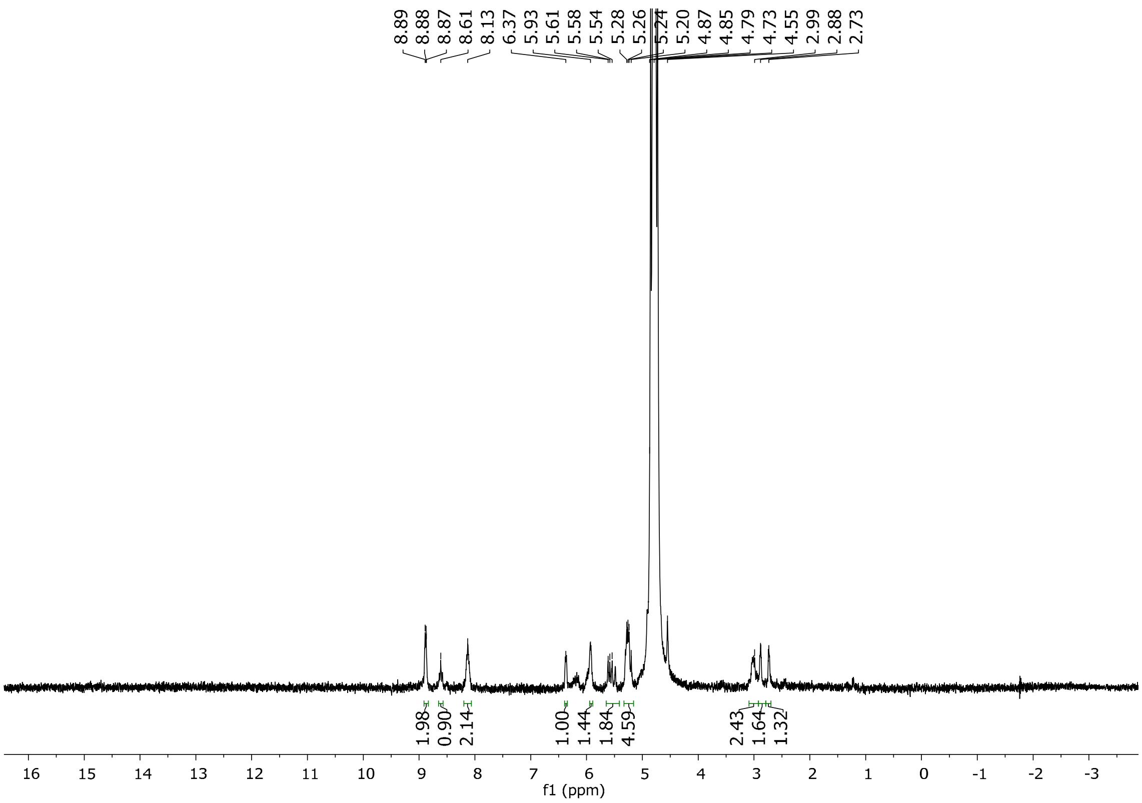

Figure S21: ^{1}H NMR spectra of photochemical transformation of 1a to 2a under continuous-flow conditions [Flow rate: 0.12 mL/min; rpm: 3; Residence time: 4h; Conversion: 49%] on the PTQ6 (Table 3 of the manuscript, Entry 1).

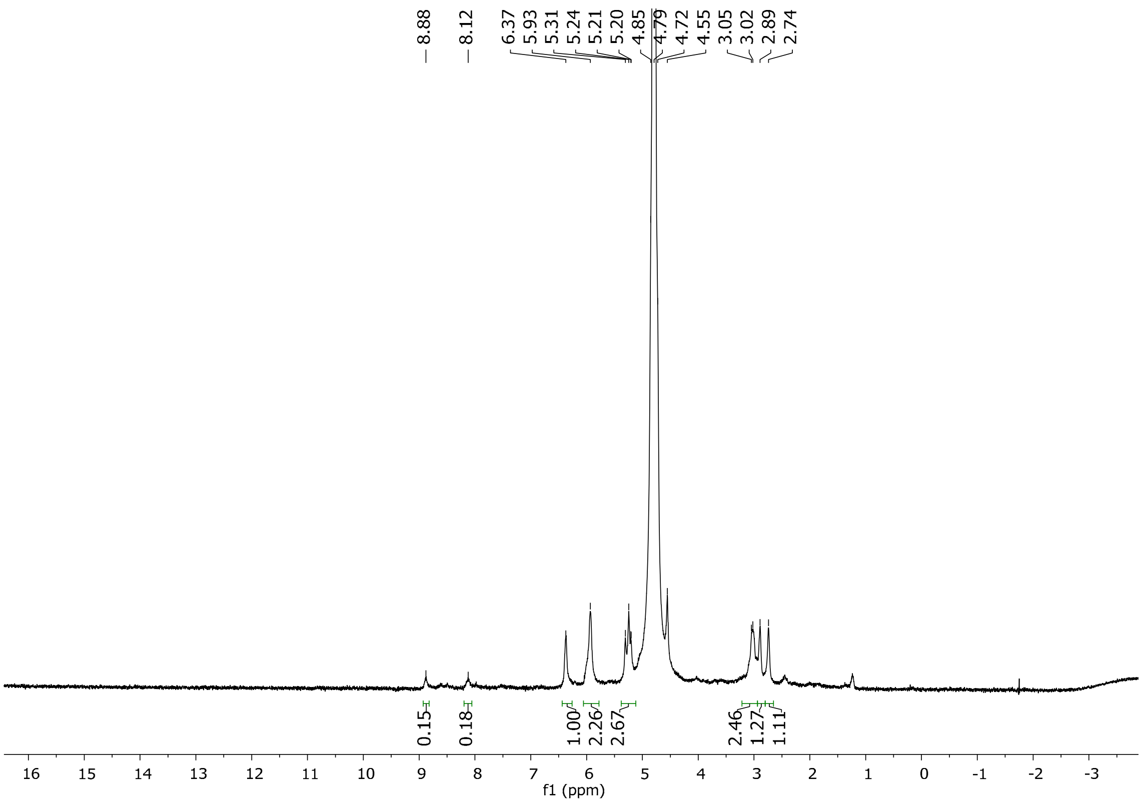

Figure S22: ^{1}H NMR spectra of photochemical transformation of 1a to 2a under continuous-flow conditions [Flow rate: 0.12 mL/min; rpm: 3; Residence time: 4h; Conversion: 92%] on the PTQ6 (Table 3 of the manuscript, Entry 2)

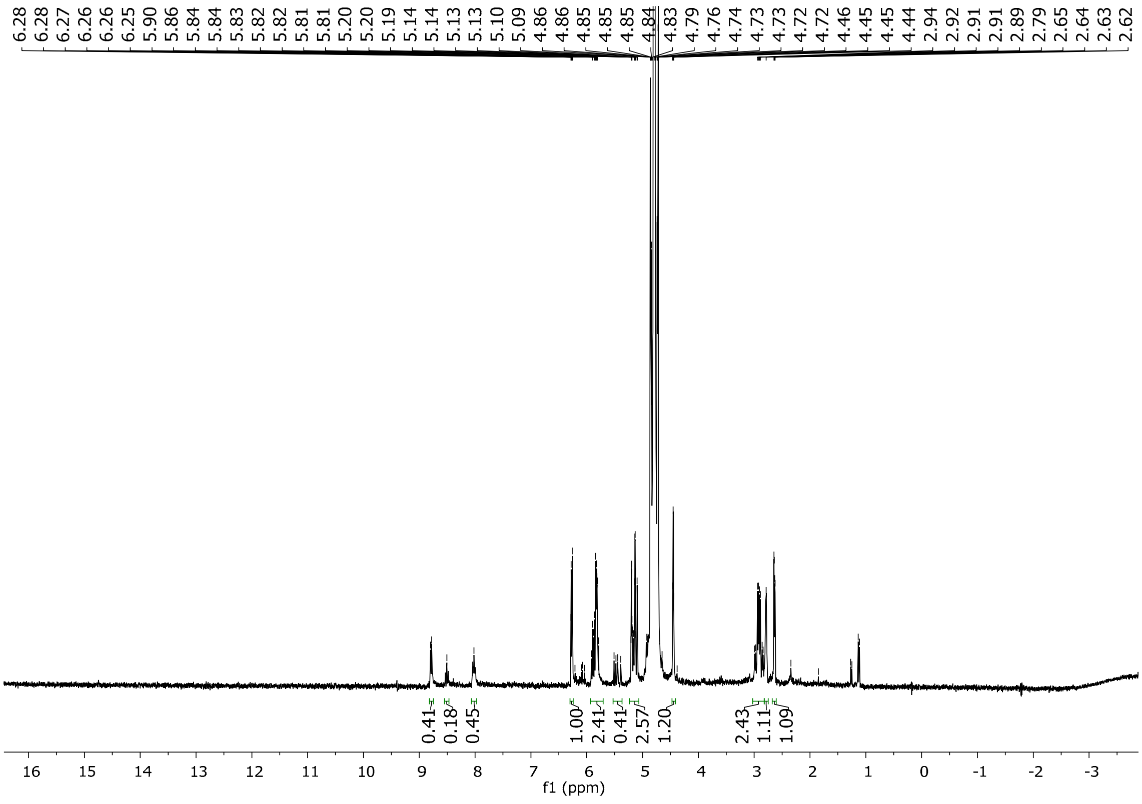

Figure S23: ^{1}H NMR spectra of photochemical transformation of 1a to 2a under continuous-flow conditions [Flow rate: 0.12 mL/min; rpm: 3; Residence time: 4h; Conversion: 83%] on the PTQ6 (Table 3 of the manuscript, Entry 2)

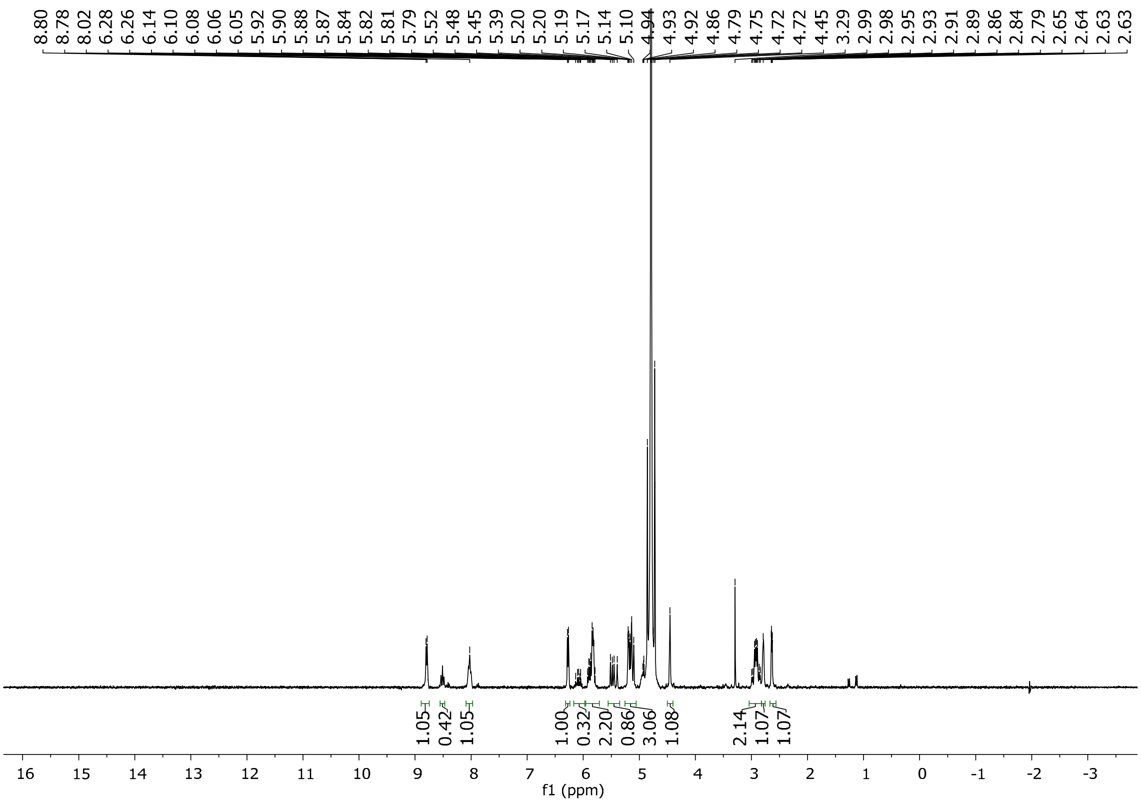

Figure S24: ^{1}H NMR spectra of photochemical transformation of 1a to 2a under continuous-flow conditions [Flow rate: 0.12 mL/min; rpm: 3; Residence time: 4h; Conversion: 66%] on the PTQ6 (Table 3 of the manuscript, Entry 4)

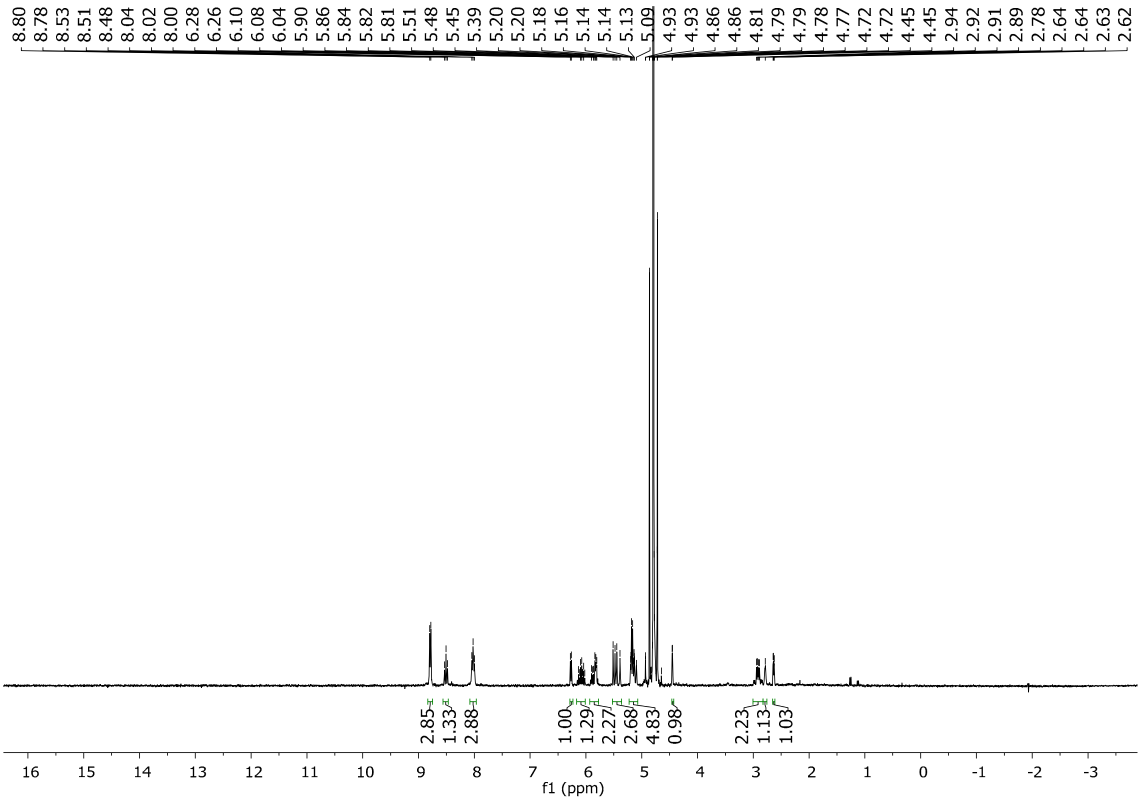

Figure S25: ^{1}H NMR spectra of photochemical transformation of 1a to 2a under continuous-flow conditions [Flow rate: 0.12 mL/min; rpm: 3; Residence time: 4h; Conversion: 41%] on the PTQ6 (Table 3 of the manuscript, Entry 5)

^{1}H NMR spectra

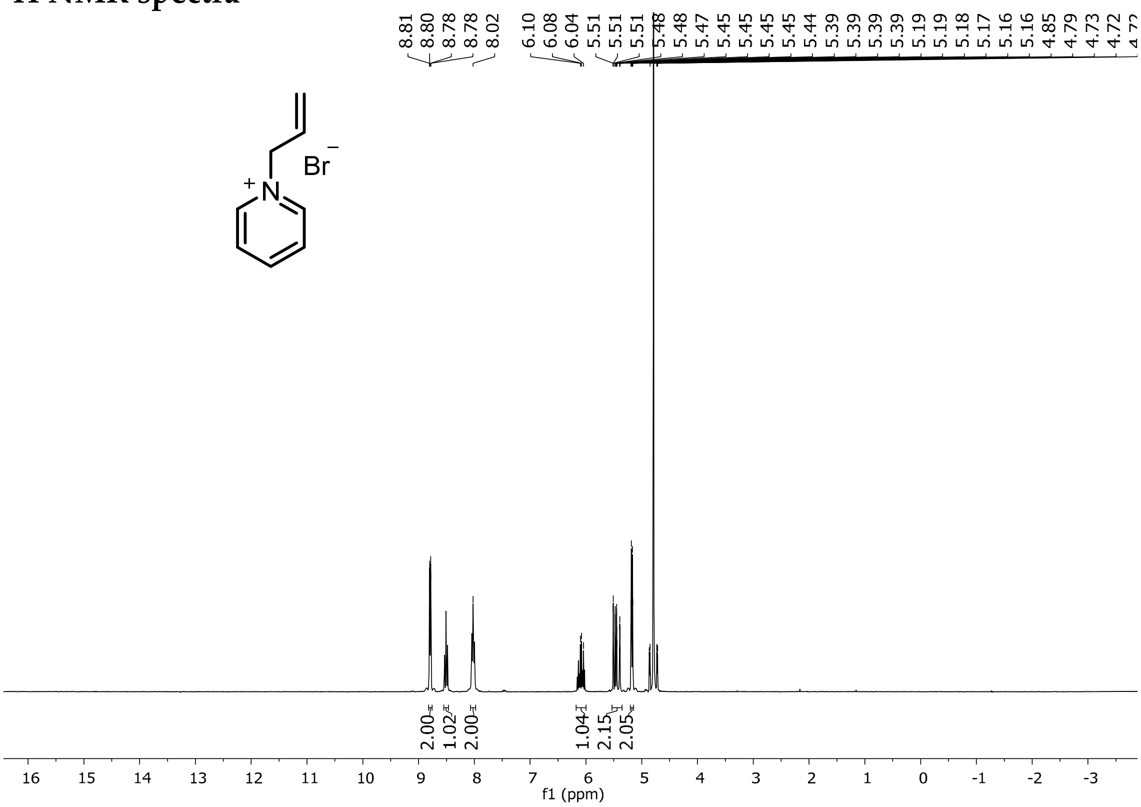

Figure S26: ^{1}H NMR spectra of 1-allylpyridinium bromide, in accordance with literature [7].

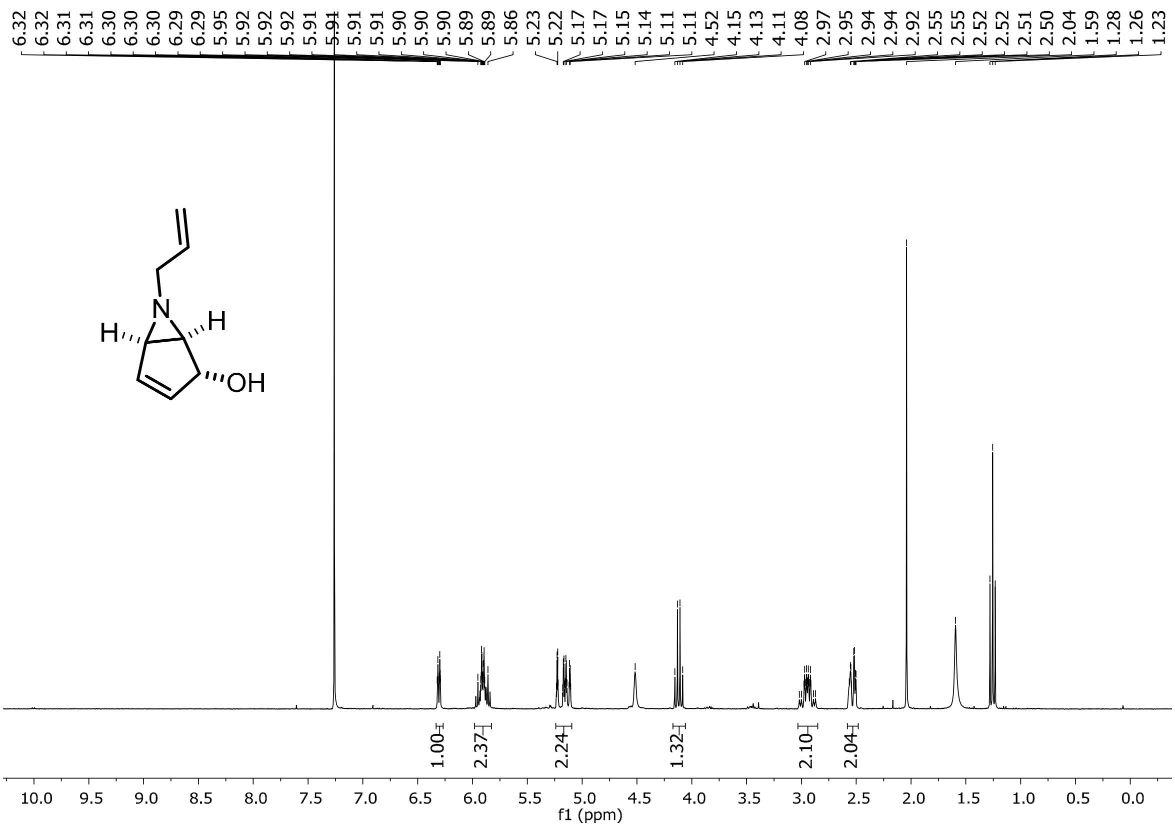

Figure S27: ^{1}H NMR spectra of 6-allyl-6-azabicyclo[3.1.0]hex-3-en-2-ol, in accordance with literature [7].
